# Three Novel Antisense Overlapping Genes in E. coli O157:H7 EDL933

**DOI:** 10.1128/spectrum.02351-22

**Published:** 2022-12-19

**Authors:** Franziska Graf, Barbara Zehentner, Lea Fellner, Siegfried Scherer, Klaus Neuhaus

**Affiliations:** a Core Facility Microbiome, ZIEL – Institute for Food & Health, Technische Universität München, Freising, Germany; b Chair for Microbial Ecology, TUM School of Life Sciences, Technische Universität München, Freising, Germany; Univeristy of Nebraska Medical Center

**Keywords:** overlapping genes, EHEC O157:H7, protein, prokaryotic genomes, competitive growth assays, phenotype, ribosome footprinting

## Abstract

The abundance of long overlapping genes in prokaryotic genomes is likely to be significantly underestimated. To date, only a few examples of such genes are fully established. Using RNA sequencing and ribosome profiling, we found expression of novel overlapping open reading frames in Escherichia coli O157:H7 EDL933 (EHEC). Indeed, the overlapping candidate genes are equipped with typical structural elements required for transcription and translation, i.e., promoters, transcription start sites, as well as terminators, all of which were experimentally verified. Translationally arrested mutants, unable to produce the overlapping encoded protein, were found to have a growth disadvantage when grown competitively against the wild type. Thus, the phenotypes found imply biological functionality of the genes at the level of proteins produced. The addition of 3 more examples of prokaryotic overlapping genes to the currently limited, yet constantly growing pool of such genes emphasizes the underestimated coding capacity of bacterial genomes.

**IMPORTANCE** The abundance of long overlapping genes in prokaryotic genomes is likely to be significantly underestimated, since such genes are not allowed in genome annotations. However, ribosome profiling catches mRNA in the moment of being template for protein production. Using this technique and subsequent experiments, we verified 3 novel overlapping genes encoded in antisense of known genes. This adds more examples of prokaryotic overlapping genes to the currently limited, yet constantly growing pool of such genes.

## INTRODUCTION

Overlapping genes are genes whose coding regions are completely or in major parts located on the same DNA locus. The double strand structure of a DNA molecule and the triplet codon periodicity theoretically allows up to 6 reading frames in a given segment. The evolution of such peculiar genes becomes possible due to the redundancy of the genetic code, i.e., most amino acids can be specified by more than one codon. Thus, each frame has the potential to code for a functional protein product in an overlapping fashion (1). However, very short overlaps, which are used as translational coupling of open reading frames (ORFs) in a polycistronic operon, are not considered as overlapping genes here. The protein parts encoded in the short overlapping areas are minor only and normally do neither form a domain, nor have a function on protein level (2, 3).

Overlapping genes are distributed across all domains of life including eukaryotes (4), bacteria (5), and viruses, whereby the genomes of the latter are commonly known for harboring large proportions of non-trivial (overlap > 30 nt) overlapping genes (6). This characteristic is hypothesized to be a result of the spatially limited viral capsid that exerts a selection pressure on the genome to encode the maximum number of proteins within a limited scope of genome length (7). However, this has been challenged by constructing decompressed viral genomes (i.e., removing all overlaps; [8]). Even though bacterial genomes are densely packed with protein-coding genes (9), long overlapping genes in bacteria have not been subject of targeted studies until recently (10). Early discoveries of such gene pairs rather represented serendipity than directed research (11–14); nonetheless, these findings called into doubt the belief that the evolutionary constraint of one mutation detrimentally affecting 2 genes simultaneously prevents the existence of overlapping genes (15). Yet, bacterial genome annotation programs often repress the annotation of non-trivial overlaps (16, 17). These circumstances have probably led to a systematic underrepresentation of such genes (18) and, supposedly, a significant portion of bacterial overlapping genes still awaits discovery. In recent years, an increasing number of studies provided strong indications for the existence of long overlapping genes in bacteria (10, 19, 20). In addition, evidence for the functionality of non-trivial overlapping genes in bacteria is accumulating (3, 21–25).

While most non-overlapping (i.e., “common”) genes are more strongly expressed and thus have been discovered, overlapping genes tend to be weakly expressed and rather short in comparison (10, 24, 26). Deep sequencing-based methods like ribosomal profiling facilitate detailed measurement of bacterial translation *in vivo* (27), and can thereby help to identify potential overlapping coding regions. In any case, the examples of established bacterial overlapping genes known to date suggest that such genes tend to be taxonomically restricted (21, 22, 24). Studies have shown that overlapping genes typically code for specialized rather than basic cellular functions (2). In addition, overlapping genes tend to have a larger degree of structural disorder compared to proteins encoded by non-overlapping genes (28) and it is assumed that overlapping genes emerge *de novo* through a process termed overprinting (29, 30). Here, a new reading frame emerges by few mutations as protein-coding within an existing (mother) gene.

In this study, we experimentally identified 3 non-trivial overlapping genes in the pathogen Escherichia coli O157:H7 (EHEC) EDL933 from originally 11 candidate genes (not shown), which caught our attention. EHEC has a genome of about 5 Mbp, a plasmid of 93 kbp, and 5772 annotated genes are known from both genetic elements. EHEC is a contributor to severe gastroenteritis in the developed world and infection leads to development of the hemolytic uremic syndrome in some individuals (31). While the bacteria are able to circulate in the environment, the main source for zoonotic infections are possibly ruminants, for which the bacterium normally behaves as commensal. EHEC are transmitted by direct animal contact, via food, dairy products, water, or the environment (32). Microorganisms show a high adaptability, which is likely attributable to their need for resilience when confronted with alternating and at times adverse environmental conditions, including antibiotics (33). Therefore, it is important to understand the evolution of novel genes in the genomic background, which contributes to a better understanding of resistance and pathogenicity during EHEC’s evolution.

## RESULTS

### Genomic localization of the overlapping open reading frames (OLOs).

The following section puts the novel OLOs into genomic context and describes their structural and regulatory elements found as described in the following sections. None of the OLOs is located in a phage-derived region.

**FIG 1:**
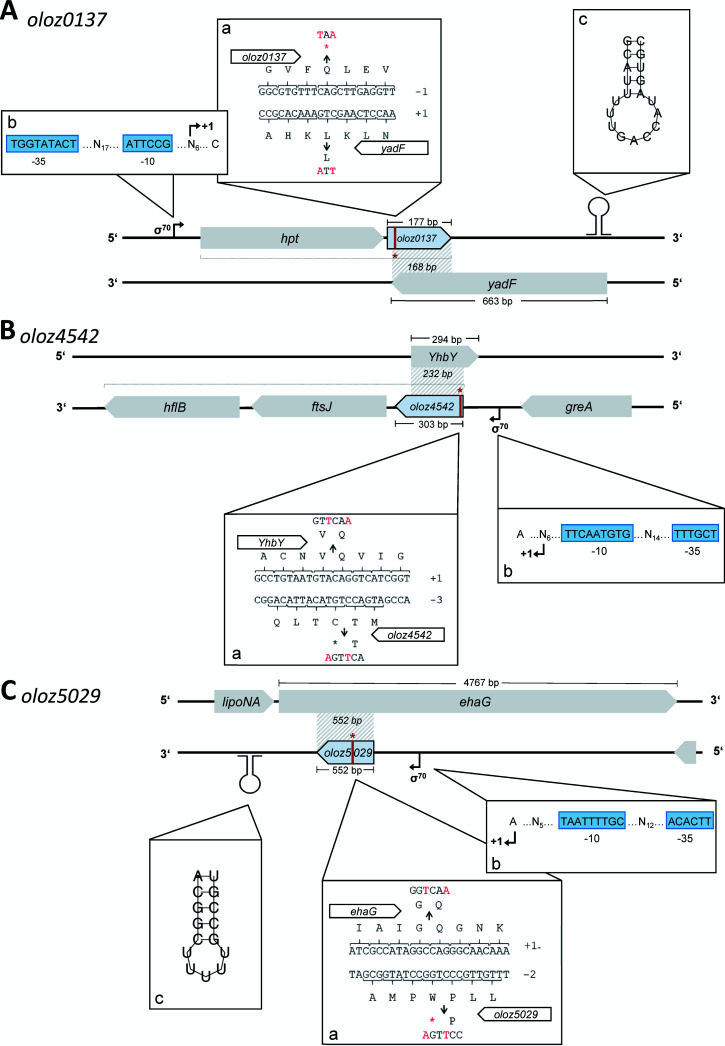
Genomic organization of the three-novel overlapping open reading frames (light blue arrows), overlapping nontrivially in antisense to annotated genes (gray arrows). The DNA is represented by black lines. (A) *oloz0137*, (B) *oloz4542*, and (C) *oloz5029*. Polycistronic transcription is indicated by dotted gray brackets. Each gene panel has 3 subpanels: (a, b, and c). (a) genomic structure of respective translationally arrested mutants. Stop codons (*) were introduced through base substitutions at positions indicated (marked by red letters). Importantly, the reading frame of the mother gene was left intact by careful choosing the mutation. Reading frames are denoted by Arabic numerals (annotated gene set to reading frame +1). (b), promoter sequence and transcription start site (TSS). Sequences of the -10 box and -35 box (blue boxes) and the TSS (transcription start site +1, black arrow) are given. N_x_ indicates the length of the spacer between the boxes as well as the distance between the -10 box and the TSS in nt. (c), secondary structures of terminator stem loops predicted by the RNAfold web server. Given that *oloz4542* is transcribed polycistronically with *ftsJ* and *hflB*, no separate terminator was specified in this case.

The novel gene *oloz0137* (Fig. 1A) has a length of 177 nt and overlaps partially in antisense with 168 nt of the annotated gene *yadF* (length, 663 nt; locus tag, EDL933_RS00665; protein product, carbonic anhydrase). The reading frame of *oloz0137* is -1 relative to its mother gene *yadF* (set to reading frame +1). Upstream of *oloz0137*’s putative start codon (ATG_146372_), the hypoxanthine phosphoribosyl transferase coding gene *hpt* (EDL933_RS00660) is found in an intergenic distance of 31 nt. Both, *oloz0137* and *hpt* appear to be co-expressed from a σ^70^ promoter, which is 76 nt upstream of *hpt*. Transcription terminates between 239 nt and 497 nt downstream of the stop codon of *oloz0137* (TGA_146546_).

*oloz4542* (303 nt) overlaps in antisense to the annotated gene *yhbY* (294 nt, EDL933_RS21645), which encodes the ribosome assembly RNA-binding protein YhbY (Fig. 1B). The 2 genes share 232 nt of their sequences. The reading frame of *oloz4542* is -2 in respect to its mother gene set to +1. The *ftsJ*-*hflB*-operon is located 55 nt downstream of *oloz4542*. It codes for the 23S rRNA methyltransferase FtsJ (EDL933_RS21640) and the ATP-dependent zinc metalloprotease HflB (EDL933_RS21635) (34). The transcriptional unit formed is polycistronic, containing *oloz4542*, *ftsJ* and *hflB*. Transcription is initiated from a σ^70^ promoter 64 nt upstream of the proposed OLO start codon (ATG_4157848_).

The sequence of *oloz5029* (552 nt) (Fig. 1C) is completely embedded in antisense in the annotated gene *ehaG* (length, 4767 nt; locus tag, EDL933_RS23925; protein product, autotransporter adhesin EhaG) (35). With respect to the reading frame of the annotated gene (set to +1), *oloz5029* is coded in frame -2. A σ^70^ promoter is located 33 nt upstream of the proposed translational start of *oloz5029* (ATG_4607745_). Transcription ends at a *rho*-independent terminator, which is 383 nt downstream of the stop codon (TAA_4607196_).

### Ribosome profiling and RNA sequencing.

Using data sets of E. coli O157:H7 EDL933 for RNA sequencing (RNA-seq) and ribosome profiling (RP), we analyzed signals concerning translation and transcription of OLOs (36). The measurements had been made using standard laboratory conditions, i.e., aerobic cultivation in LB medium at 37°C and cells had been harvested at the transition into the early stationary phase.

**FIG 2:**
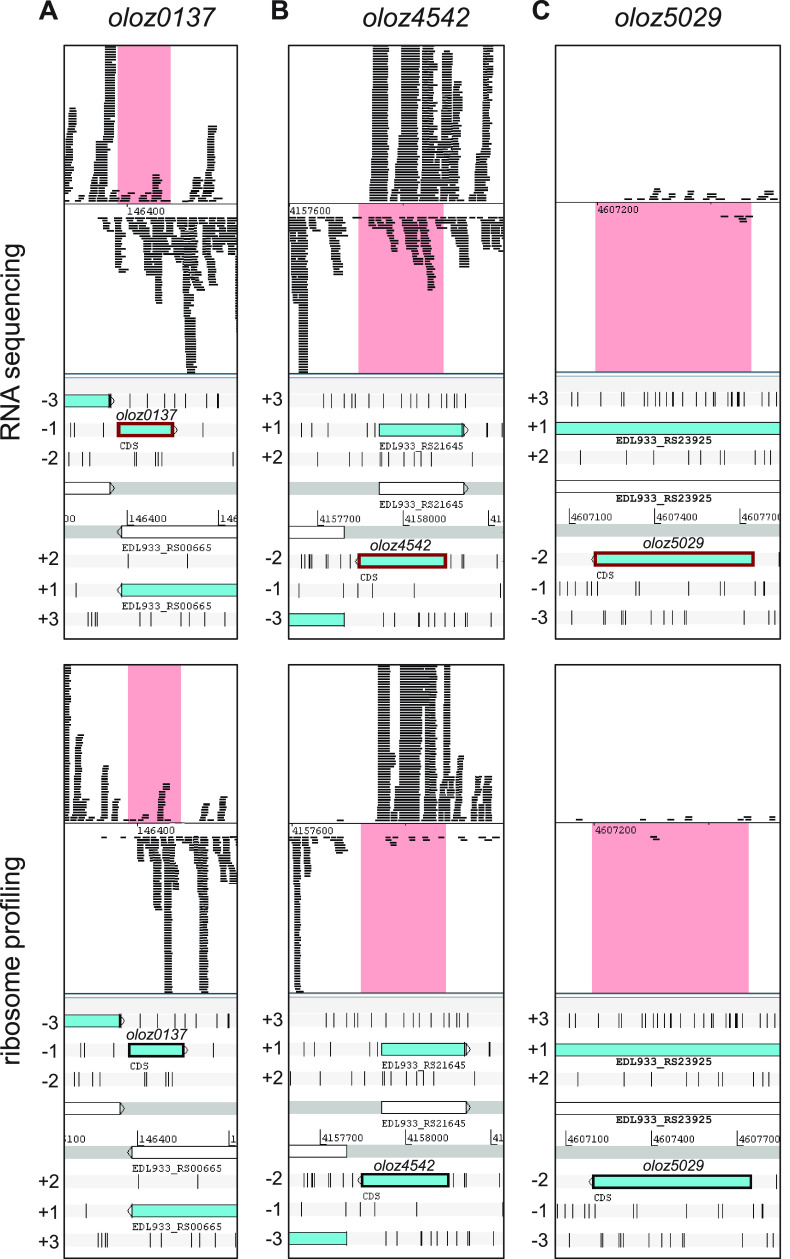
Transcription and translation of the putative overlapping genes. Top panels, transcription (RNA sequencing); bottom panels, translation (ribosome profiling). E. coli O157:H7 str. EDL933 was cultivated under standard conditions (LB, 37°C) and harvested at the beginning of the early stationary phase. The sum signal of 2 biological replicates are visualized using Artemis 17.0.1 (101). The novel genes are shown with a bold line and the corresponding reads are highlighted in pink. Reading frames are given relative to the annotated mother gene which is defined as frame +1. (A) *oloz0137*, (B) *oloz4542*, and (C) *oloz5029*.

Both RNA-Seq and RP show signals for transcription and translation, respectively, in regions overlapping annotated genes (Fig. 2). The signals were evaluated using reads per kilobase gene length per million sequenced reads (RPKM) values, i.e., reads normalized to gene length and sample sequencing. The novel protein-coding reading frames were designated *oloz0137*, *oloz4542*, and *oloz5029.* Concerning *oloz0137*, RPKM values of 23.7 and 14.9 were found for RNA-seq and RP, respectively. Next, *oloz4542* had RPKM values of 44.6 and 4.7, respectively. The third open reading frame, *oloz5029*, had RPKM values of only 1.0 and 0.3, indicating very weak expression. Nevertheless, the last gene was taken into account because further experimental investigations provided clear evidence for a protein-coding nature also for this ORF (see following sections).

### qPCR verifies transcripts and gene regulation.

**FIG 3:**
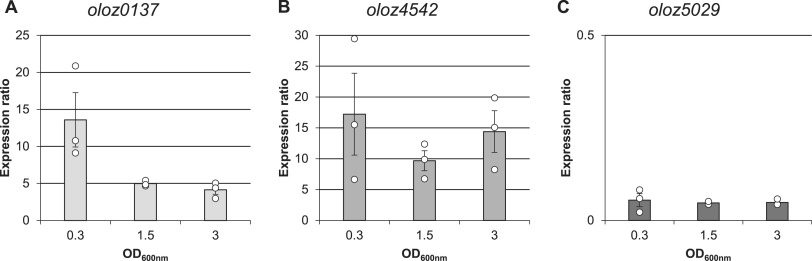
Transcription of *oloz0137*, *oloz4542* and *oloz5029* at different growth phases. qPCR was used to measure the expression ratio for each overlapping gene above *cysG*. The latter gene was used for normalization of the Δcq values, i.e., showing the difference in threshold cycles for the novel genes relative to *cysG*. EHEC were grown in LB and harvested at OD_600_ = 0.3 (early exponential phase), OD = 1.5 (late exponential phase) and OD = 3.0 (stationary phase), respectively. Mean value and standard deviations of three biological replicates were calculated and are shown as error bars. (A) *oloz0137*, (B) *oloz4542*, and (C) *oloz5029*.

Quantitative PCR (qPCR) was used to verify native transcripts for each OLO in EHEC and to monitor mRNA expression levels throughout different growth phases. Transcripts were quantified relative to *cysG* mRNA levels. The *cysG* gene is coding for a multifunctional uroporphyrin III C-methyltransferase involved in siroheme synthesis and has a near constant transcription level in E. coli (37). Transcripts for each OLO at every time point examined were detected. The RNA quantification relative to *cysG* revealed an over-time decline in expression of *oloz0137* (Fig. 3A). The expression of *oloz4542* fluctuated throughout the different growth phases (Fig. 3B). The copy number of *oloz05029* mRNA was low and remained unchanged over time (Fig. 3C). At this point, it should be mentioned that the quantitative analysis could be biased by asRNAs, which could prime retro-transcription events independently from a specific DNA primer (38), but see below for further discussion.

### Promoters and transcription start sites.

**FIG 4:**
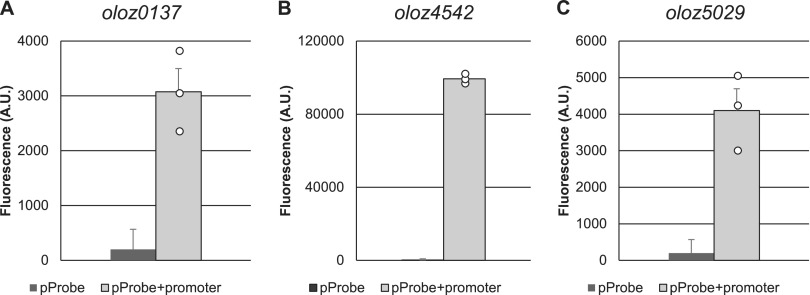
Promoter assays using GFP. Putative promoter sequences of each overlapping open reading frame (OLO) were cloned upstream of the GFP gene in pProbe-NT. An empty plasmid was used as a negative control. GFP expression was measured in E. coli DH5α after cultivation in LB at OD_600nm_ = 0.6. Mean fluorescence intensity (A.U., arbitrary units) of 3 biological replicates are shown; error bars indicate standard deviations. The statistical significant difference between the empty vector and the promoter construct was confirmed with a Welch two sample *t* test (*P* ≤ 0.01). (A) *oloz0137*, (B) *oloz4542*, and (C) *oloz5029*.

Upstream sequences for each overlapping gene containing the supposed promoter (Prom) were cloned into a promoter-less green fluorescent protein (GFP) reporter plasmid (pProbe-NT) for testing. Expression of GFP is induced from the promoter sequence inserted and is measured by fluorescence intensity. Here, permissive E. coli DH5α were transformed with the resulting vector constructs and cultivated in LB. The empty vector was used as a negative control. Cells with pProbe-NT-Prom*_oloz0137_* produced the least fluorescence signal of all 3 promoters tested, which was about 15-fold higher compared to the control (Fig. 4A). We also observed significantly increased fluorescence in cells carrying Prom*_oloz5029_* (20-fold higher) (Fig. 4C). The strongest promoter activity was measured for *oloz4542*, which was about 494-fold higher compared to control. While we would have expected for *oloz5029* a weak promoter activity based on RNA-Seq and RP results, the weak signal for *oloz0137* was rather surprising. In any case, all promoter regions tested produced a fluorescence signal significantly above control.

Transcription start sites (TSSs) were determined via Cappable-seq (39) using experiments conducted by Zehentner, Ardern (40). The TSS expression strengths matched the trend of the promoter assay results: the TSS-expression data identified a weak TSS upstream of both *oloz0137* and *oloz5029*, respectively. Since, for the former, almost no expression was detected, we checked the TSS of the next gene upstream, *hpt*. Indeed, a strong TSS was found (see below). Concerning the overlapping genes, *oloz4542* had the by far highest read density; its upstream region was found to harbor 2 strong transcription start sites directly adjacent to each other, which fits well to the promoter assays above (Table 1).

**TABLE 1:** Expression strength of transcription start sites^*a*^

Gene	TSS genome position	Expression strength
		$\frac{\mathrm{reads}\;\mathrm{TSS}\;*\;1000}{\mathrm{mapped}\;\mathrm{reads}}$
*oloz0137*	146133	0.9
*hpt* (upstream gene)	145733	471.3
*oloz4542*	4158211	60.6
	4158212	98.8
*oloz5029*	4607779	2.5

a Expression strength of the transcription start site (TSS) was determined with Cappable-seq.

**FIG 5:**
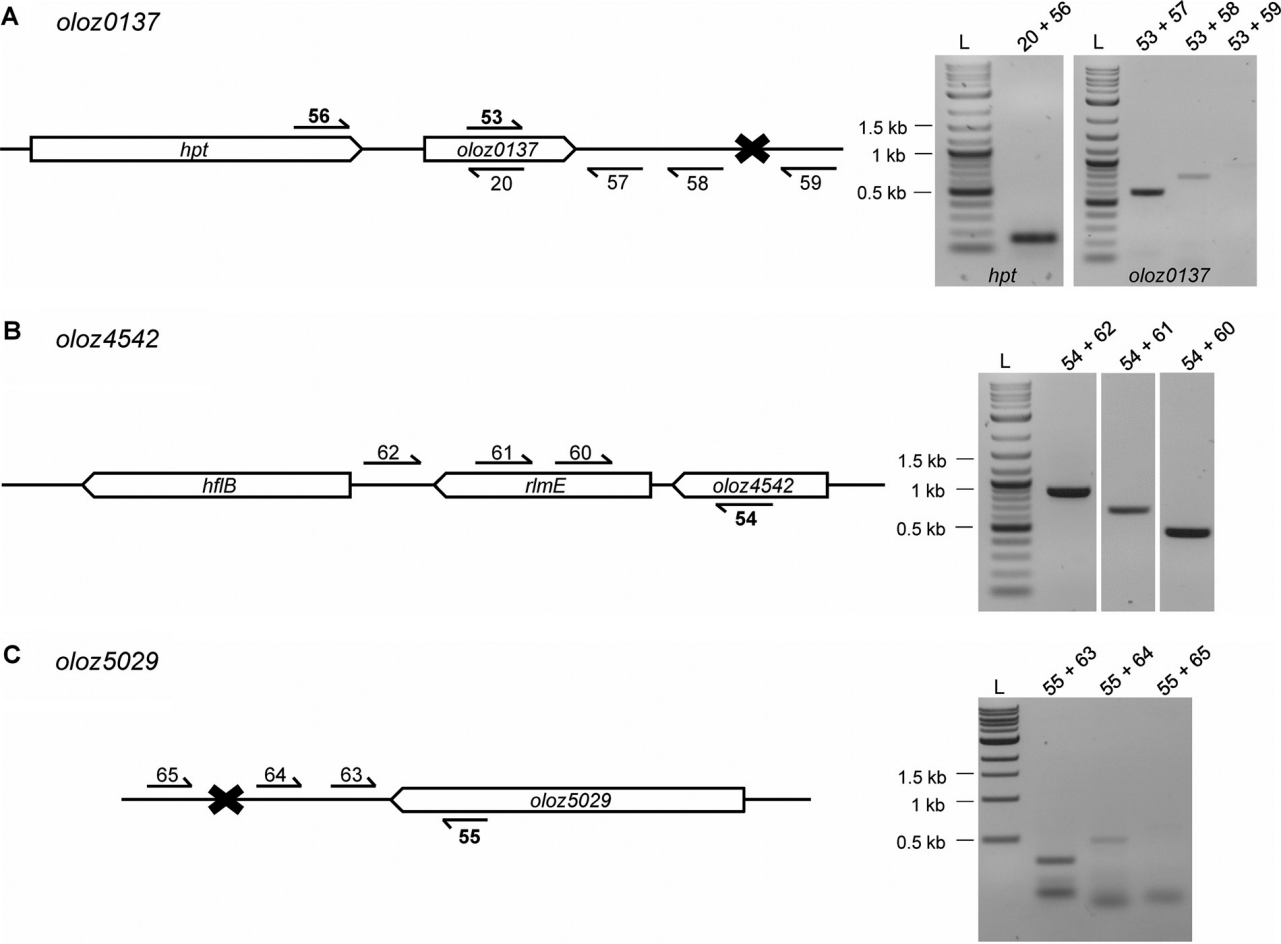
Transcript analysis of each overlapping gene using RT-PCR. To the left, primer positions are indicated for each gene. To the right, agarose gel analysis of the PCR products is shown. If the relevant bands were located on different gels, they are displayed true to scale relative to each other. mRNAs were reverse transcribed to cDNA with specific reverse primers (i.e., 20, 57, 58, and 59; 62, 61, and 60; 65, 64, and 63, respectively). Subsequent RT-PCRs were conducted with each reverse primer used and a gene specific forward primer (i.e., 56 or 53, 54, and 55, respectively; bold) as indicated. A PCR product is generated only if a transcript of at least the length covered by both primers is present. Numbers above each lane of the gels indicate the specific PCR. The respective negative controls (not shown), i.e., samples without reverse transcriptase, showed no bands whatsoever on the gel. L, 1 kb (+) ladder (NEB). (A) *oloz0137* and *hpt*, (B) *oloz4542*, and (C) *oloz5029*.

Subsequently, putative promoter sequences found in the TSS data above were inspected bioinformatically using the bacterial promoter prediction program BProm (41). The program was applied to analyze the highest scoring TSS experimentally found for predicted σ^70^ promoters. It identified suitable sequences for *oloz4542* and *oloz5029* having linear discriminant function (LDF) scores of 2.95 and 1.86, respectively (an LDF of ≥ 0.2 is considered to be significant). For both genes, the experimental data, in combination with the bioinformatics prediction, provided a coherent picture. Each promoter has the typical distance of around 7 nt (42) between the TSS and the -10 box (*oloz4542*: 6 nt, *oloz5029*: 5 nt). However, for *oloz0137*, no appropriate sequence with acceptable distance between the TSS and the -10 box was found. Even though the discrepancy of transcriptome and translatome data could be a result of convergent transcription, the lack of a matching promoter, low TSS expression levels, and weak promoter activity led us to the conclusion that *oloz0137* forms a polycistronic transcript along with *hpt*. Indeed, a transcript spanning both genes was detected via reverse transcription-PCR (RT-PCR) (Fig. 5). We therefore screened the upstream region of *hpt* for TSS and promoter sequences and successfully identified a strong TSS alongside (as described above) with a compatible promoter with an LDF score of 4.52 and 6 nt distance between TSS and -10 box.

### Analysis of mRNA for transcription termination determination.

RT-PCR was performed to narrow down transcript boundaries of the novel genes. Toward this end, EHEC RNA was reverse transcribed to cDNA using gene specific reverse primers binding either down- or upstream of the terminator (Fig. 5). The resulting cDNA was subsequently used as template in a PCR that involved each the same reverse primer and a forward primer binding within the coding region of the gene tested. However, if the reverse primer was located upstream of each terminator, a cDNA fragment was synthesized to which both primers of the subsequent PCR could bind. The same principle was applied to test for polycistronic transcription concerning *oloz0137* and *oloz4542* (forward primer binds in putative polycistronic partner gene, reverse primer binds in the OLO). The results for the individual OLOs were as follows: *oloz0137*’s transcription is terminated between 239 nt and 497 nt downstream of its stop codon. Besides, the RT-PCR revealed that *oloz0137* is transcribed polycistronically with *hpt* (Fig. 5A). *oloz4542* does not appear to have a terminator of its own, but forming an operon with the downstream genes *hflB* and *rlmE*. The RT-PCR product received is evidence for an mRNA spanning from *oloz4542* to *rlmE* and not ending upfront the start codon of *hflB* (Fig. 5B). We conclude that *oloz4542* forms a transcriptional unit together with *hflB* and *rlmE*, with the terminator of *hflB* defining the end of the polycistronic mRNA. Finally, the terminator of *oloz5029* was located between 210 nt and 346 nt downstream after the stop codon (Fig. 5C). A stem-loop structure was discovered in this region, which we assume to represent the *rho*-independent terminator of this gene.

### Overexpression and Western blots.

The protein-coding capacity of the OLOs was verified by Western blots. Overexpression of the proteins was performed in E. coli Top10 for each gene individually. The putative coding sequence was cloned in-frame with a C-terminal sequential peptide affinity (SPA) tag (7.7 kDA) into the plasmid pBAD-SPA (43). Following separation of whole cell lysates on tricine gels, fusion proteins carrying the C-terminal tag were visualized using a SPA-specific antibody. Samples were taken at different time points after induction. SPA-tagged glutathione *S*-transferase (*gst*::SPA, 23.7 kDa + 7.7 kDa) was used as positive control.

**FIG 6:**
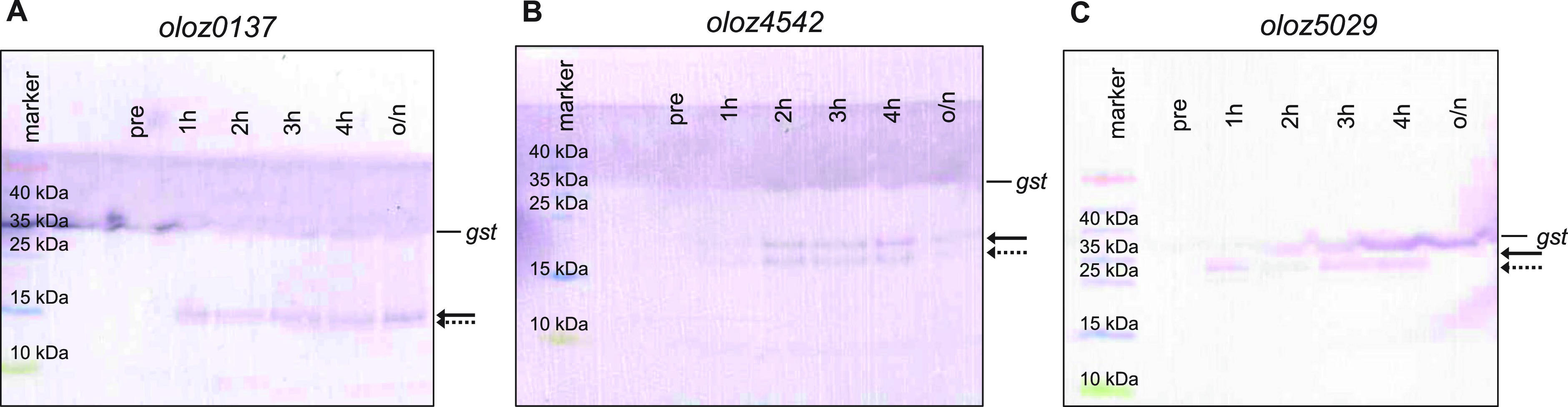
Western blots of protein products. Proteins were expressed in frame, fused C-terminally to an SPA-tag from a pBAD-plasmid, in E. coli Top10. Cells were grown in LB and harvested before induction (pre) as well as 1, 2, 3, and 4 h, and overnight (o/n) after induction. Harvested sample volume was adjusted in order to obtain equal cell numbers. Proteins were separated on 16% tricine gels. Marker, PageRuler Prestained Protein Ladder (Thermo Fisher Scientific); *gst*, positive control of glutathione *S*-transferase, the original control lane is not displayed as the *gst*-band showed across the entire gel; Bold arrows point out the respective full-length product, dashed arrows show the smaller proteins. (A) *oloz0137*, (B) *oloz4542*, and (C) *oloz5029*.

Western blotting indicated that all 3 genes have the potential to encode a stable protein product. The SPA-tagged proteins of *oloz0137* (6.1 + 7.7 kDa) (Fig. 6A), *oloz4542* (10.9 + 7.7 kDa) (Fig. 6B), and *oloz5029* (19.1 + 7.7 kDa) (Fig. 6C) appeared at the expected size. In addition to the full-length protein band, in each case, a lower band could be observed. For *oloz0137*, the lower band migrated at approximately 12 kDa. For the tagged protein product of *oloz4542*, a shorter fusion protein was detected at a length of about 14.2 kDa. Interestingly, the sequence of *oloz4542* harbors an alternative start codon that would result in a protein product of 7.7 kDa length (Fig. S1). Together with the SPA-tag, a theoretical size of 15.4 kD would be expected, which matches well to the observed band at 14.2 kDa. In addition, the Western blot for *oloz5029* showed a shorter protein product corresponding to about 25 kDa. In any case, each of the shorter proteins observed could also be degradation products instead of true isoforms.

### Putative protein structure.

To gain more insight about the OLO protein products, protein structure and function were predicted bioinformatically with PredictProtein (44) (Fig. S2). Firstly, for *oloz0137*, *alpha*-helix and *beta*-strand structures were located along the entire length of the protein, but 58% of the protein were predicted to be disordered. A signal peptide was predicted at the N-terminus. The protein of *oloz0137* was assigned to the inner membrane. Secondly, *oloz4542* appears to have several *alpha* helices and *beta* sheets and only 26% disordered regions. Three putative disulfide bonds were detected, each potentially connecting amino acids 3 with 69, 40 with 70, and 63 with 72. The protein topology is described as mainly cytoplasmic, but forming a putative transmembrane region for amino acids 69 to 86. The amino acids 87 to 100 are predicted as extracellularly located. In any case, the protein of *oloz4542* appears to be secreted. Finally, the secondary structure of *oloz5029* mainly consists of *alpha* helices with few *beta* sheets. The protein was found to have only few disordered regions (about 6%). The topology was predicted as a regular repetitive pattern, being extracellular - transmembrane - cytoplasmic - transmembrane - extracellular. The protein product of *oloz5029* was assigned to the inner membrane. Additionally, protein structures were predicted with AlphaFold (45) (Fig. S3). The results suggest that protein products of *oloz0137* and *oloz4542* are structured as mainly alpha helices. For *oloz5029*, AlphaFold predicted a completely disordered protein structure with very low confidence based on the small number of reference examples.

### Growth phenotypes indicate functionality of protein products.

Translationally arrested mutants were created to test for the functionality of the putative OLOs. The major distinguishing feature between the translationally arrested mutants and the wild-type strain were 2 chromosomal point mutations each that introduced a premature stop codon in the OLO, while leaving the reading frame of the mother gene intact, allowing for the encoding of the unchanged protein. To investigate the effect of the translational arrests on growth behavior of each strain, the wild type and mutant strains were grown in competitive growth assays. In previous experiments, wild type and mutants were cultivated individually under exact same conditions. In comparison to the wild-type strain, mutants grew slightly delayed especially in the lag phase, thereby providing the first indication of a differing growth behavior (Fig. S4).

**FIG 7:**
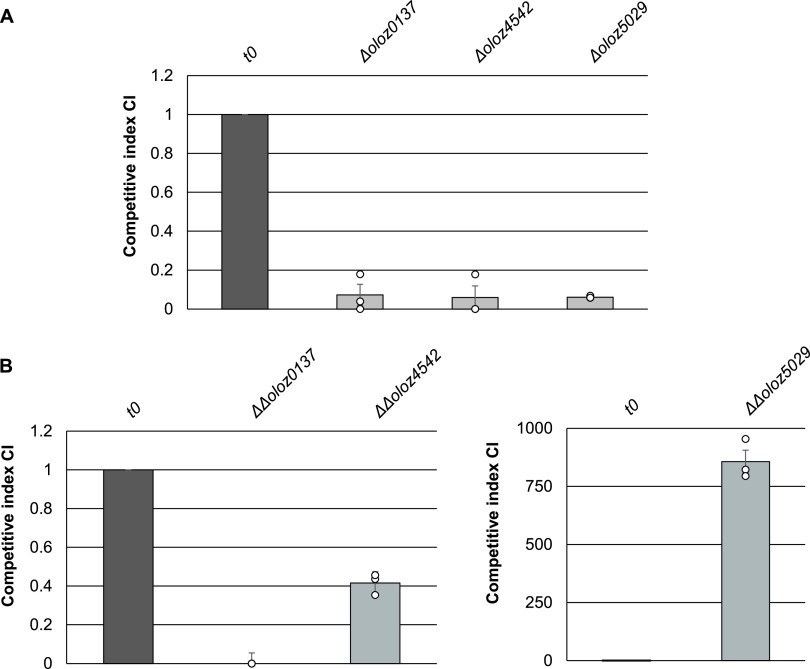
(A) Competitive growth of EHEC wild type against translationally arrested mutants (Δ*oloz0137*, Δ*oloz4542* or Δ*oloz5029*) in LB at 37°C. (B) Complemented competitive growth of EHEC translationally arrested mutants. Competitive growth with overexpression of either mutant (ΔΔ*oloz0137*, ΔΔ*oloz4542* or ΔΔ*oloz502*) or wild type OLO from pBAD/myc-HisC in LB at 37°C. Competitive indices (CI) represent the ratio of the relative abundance of each mutant carrying the mutant plasmid after 18 h of incubation regarding to the mutant over wild type plasmid input ratio at t0. Mean values of 3 biological replicates are shown and standard deviations are depicted as error bars.

Competitive growth assays, i.e., growing wild type and mutant together, are extremely sensitive since even minor differences in fitness can be distinguished (46, 47). Equal amounts of translationally arrested mutant and wild type were used to inoculate plain LB (*t* = 0 h). Further samples were taken after 18 h of aerobic incubation. The amount of wild type and mutant in the samples was determined by Sanger sequencing. The fluorescence peaks heights at the mutated positions of each gene of interest were used to calculate the competitive index (CI) that represents the relative amount of mutant over wild type. The proportion in the inoculum is 1:1, which results in a CI of 1. After 18 h of aerobic growth in LB at 37°C, the CI values of each translationally arrested mutant was below 0.2, indicating a growth disadvantage of the mutants compared to the wild type (Fig. 7A). This indicates an unknown functionality of each overlapping gene since the amino-acid encoding capacity of each mother gene was kept the same. To validate the observed effect, we conducted competitive growth experiments with plasmid complementation of the gene translationally arrested. If the translational arrest of the OLOs causes the growth disadvantage, this should be complemented by ectopic expression of OLOs. The translationally arrested mutants were transformed with the appropriate wild type or mutant sequence downstream of an arabinose-inducible promoter on plasmid pBAD/myc-HisC. Both strains (i.e., mutant with plasmid with the arrested gene and mutant with plasmid with the wild-type gene) were grown competitively against each other, while protein expression was induced with arabinose. For *oloz0137* and *oloz4542*, the functional protein product from the intact frame was able readily to complement the translational arrest of the chromosomal mutant. Here, the wild type gene complemented mutant strain had a pronounced growth advantage compared to the mutant strain carrying the mutant plasmid (Fig. 7B). However, an opposite effect was observed for *oloz5029*, where the complemented strain had a significant growth disadvantage compared to the mutant. This could be a result of toxicity caused by overproduction and accumulation of an otherwise low-level protein. Such high concentrations of enzymes or other functional proteins may damage cells by disturbing specific pathways or regulation, by aggregation, by distorting protein complex equilibria or cell liquid phase separation (48–52). Eventually, the protein overexpression (of this otherwise low-level expressed protein) can overburden a cell simply by depleting resources required for protein production (53, 54). Thus, the experiment was twisted. In another experiment, we grew the mutant strain with the wild type overlapping gene (i.e., EHECΔ*oloz5029 *+* *pBAD-*oloz5029*) competitively against the mutant strain (EHECΔ*olo5029*) carrying pBAD with the inverted sequence of *oloz5029*. After 18 h of competitive growth, only strain with the inverted gene sequence remained detectable. We assume overexpression of the original *oloz5029* frame to be toxic (i.e., more burdensome) indeed. The inverted frame also produced a protein (fragment) of the same length, but this appeared to be a lesser burden in comparison to *oloz5029* expression.

### Taxonomic distribution.

**FIG 8:**
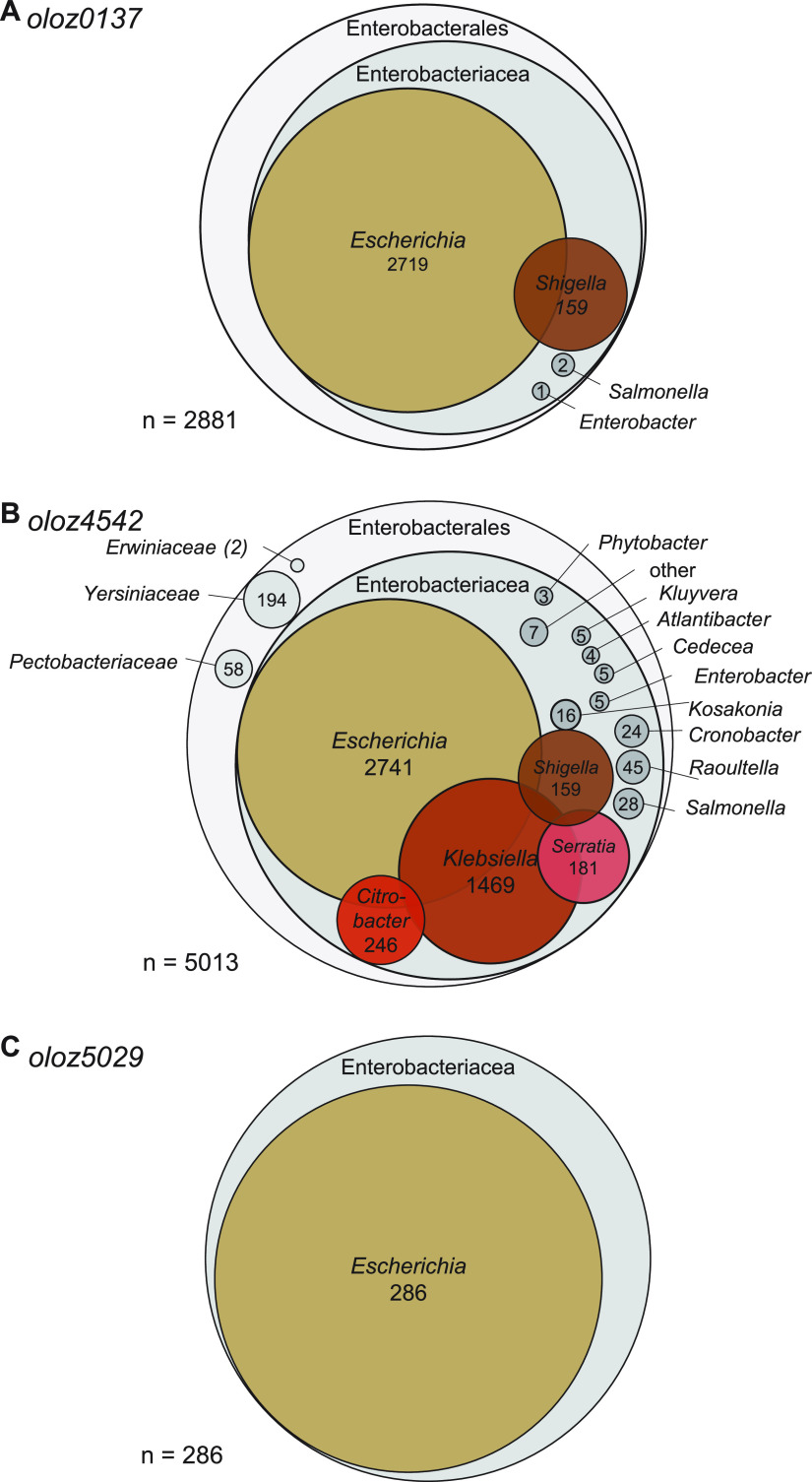
Taxonomic distribution of *olo*-homologs. tblastn was used to query the NCBI nt/nr database for homologs of the overlapping genes *oloz0137*, *oloz4542* and *oloz5029* (minimum 70% identity).

In order to resolve the taxonomic distribution of the OLOs, homologs were searched using the basic local alignment search tool BLAST (55), specifically tblastn, aligned and processed to a tree using Neighbor-joining (Fig. S5). This algorithm aligns a protein sequence to the six-frame translations of nucleotide data. A total of 2881 homologous sequences (minimum 70% identity) were found for *oloz0137*, 5013 for *oloz4542*, and 286 for *oloz5029* (Fig. 8). However, many hits were only fragments of the query sequence. For *oloz0137* and *oloz4542*, the largest proportion of homologs was found within the family *Enterobacteriaceae*. Among these, full-length reading frames (i.e., ≥ 90%) of homologs for *oloz0137* and *oloz4542* were confined to the Escherichia/Shigella clade. Outside of Escherichia/Shigella, the sequences received from the blast algorithm are fragmented. The BLAST search results for *oloz5029* revealed a clear taxonomical restriction. Homologs found were only matching to Escherichia/Shigella spp., except for a single homolog detected in Salmonella. Nevertheless, full-length sequences (≥ 90%) were detected only in Escherichia/Shigella. All overlapping gene sequences found are provided as fasta (Data set S1, S2, and S3; stored as csv for technical reasons). Concerning the mother genes, we always found a wider distribution, but then without the overlapping gene in question. Similar observations have been made before concerning other overlapping genes (22, 56).

## DISCUSSION

The existence of overlapping genes in prokaryotes has been controversial for a long time, despite the very first organism to be sequenced in its entirety was a phage containing a number of overlapping genes (57). Bacterial viruses are in constant gene flow with their bacterial hosts (58–61) and, therefore, overlapping genes in bacteria could have been a possibility to consider. However, overlapping genes were believed to be an oddity of viruses for (i) their assumed need to compress their genome, (ii) the believed difficulty of naturally finding two functional proteins in sequence space encoded at the same locus, and (iii) the question of evolution, since a single mutation would hit 2 coding genes. In any case, the need for genome compression has been disproven (7). The question of finding 2 interlaced sequences forming suitable proteins is not fully solved, but this seems to be more a matter of the genetic code, which - as it happens - generally appears to be suitable for such undertakings (1, 62–65). Finally, the question of evolvability could be turned around, since a given reading frame “protects” the other frame overlapping (66, 67). While those arguments might or might not be valid, more importantly, it seems to be a general mind-set, which does not allow overlapping genes. For instance, the annotation rules for prokaryotic genomes at NCBI explicitly forbid 2 genes at the same locus until 2021 (68). This belief seems to be some remnants of the original hypothesis “one gene - one protein - one function” according to Beadle and Tatum (69). It appears to us that this paradigmatic hypothesis was (unconsciously) expanded to “one locus - one gene - one protein - one function”. In any case, overlapping genes have been shown to exist in bacteria (1, 10, 70), and here we present evidence for the existence of three further, previously unknown overlapping genes in the genome of EHEC EDL933. We were able to detect native transcripts of all three genes using RNA-seq, RP, qPCR, and RT-PCR (Fig. 2, 3, and 5). The mRNA is expressed throughout different growth phases. Further, translation into a stable protein product was shown for each ORF using SPA-tagged proteins in Western blots (Fig. 6). In addition, we found phenotypes in competitive growth experiments for each mutant. In our experiments, only the (novel) overlapping genes of interest in each locus had been translationally arrested by inserting mutations, which create a stop codon in the novel OLO but preserve the reading frame of the mother gene (Fig. 1). Bioinformatic prediction methods, combined with experimental determinations of each TSSs (Table 1), terminators, and experiments concerning transcript lengths (Fig. 5) allowed us to elucidate the genomic structure (Fig. 1).

### Are the overlapping loci RNA or protein-coding genes?

A possible objection to our results would be that the loci just express non-coding (nc)RNA and not mRNA. As such, ncRNAs mostly exert their effects through base-pairing with another target RNA molecule (i.e., siRNA, miRNA) (71) that gets altered in its regulatory secondary structure (72) or label the target mRNA for degradation (73). Besides, *cis*-acting sRNAs and asRNAs can control protein expression by either (un)masking the ribosome binding site or by hybridizing with complementary mRNA and thereby blocking translation (74–76). However, we present strong evidence for translation of each OLO, since RP signals are only achieved for mRNAs. RP signals can be detected for the OLOs not only in the organism investigated in this study (i.e., EHEC EDL933), but also in EHEC Sakai (77) (Fig. S6). RP signals in Sakai indicate rather low expression, especially for *oloz5029*, yet this does not necessarily mean the OLOs cannot seriously affect bacterial fitness. There are examples of weakly expressed essential and non-essential proteins that were found to be detrimental for bacterial fitness, e.g., PrmC and MreD or ArpA and YbfK (78, 79). It has been pointed out that some structured mRNAs might produce erroneous RP signals (80); however, the mRNAs in question presented here do not form extensive secondary structures (data not shown). Further, as said above, a stable protein could be expressed. Besides, we show that each OLO either possesses elements and structures (TSS, promoter, terminator) that are typical for protein-coding genes itself or as part of a polycistronic mRNA. Nonetheless, the strongest indication that the novel OLOs are coding for proteins are the results of the competitive growth assays using translationally arrested mutants (Fig. 7A) including the successful complementation and, furthermore, the clear effect ectopic protein overexpression had on growth behavior (Fig. 7B). The introduced substitutions are not expected to substantially disturb any functionality of a supposed ncRNA primarily resulting from base-pairing involving many base pairs (81), but causing disruption of the overlapping encoded proteins responsible for the phenotype.

### Could the observed phenotypes be a consequence of disruption of the annotated gene?

One must consider that the phenotypes observed in the competitive growth experiments could also be a consequence of the mutation interfering with the annotated mother gene’s functionality. Yet, we consider it very unlikely that such a pronounced effect would occur simultaneously in 3 independent cases. A potential negative influence on the annotated gene could be a reduced expression of the protein product caused by codon usage bias, i.e., that the synonymous mutation introduces a codon that is not used with equal frequency as the original codon. However, none of the introduced codons is a rare codon (82–84). Ballard and Bieniek (85) investigated the effect of so-called “unpreferred” compared to “preferred” codons on gene expression in E. coli. For this purpose, they constructed a *beta*-lactamase (*bla*) allele entirely composed of unpreferred codons. Surprisingly, there was no significant change in protein abundance when comparing wild type and mutant genotype and, of particular note, no significant changes in cell density. Given that even such severe alterations of a gene do not measurably affect translation or growth, it is rather unlikely that the minor synonymous codon substitutions, as used here, cause the observed strong phenotypes.

### Putative protein function.

In case of unknown proteins, homology searches (e.g., BLAST) and bioinformatic protein structure and function prediction can provide hypotheses concerning novel genes (44).

We found that *oloz0137* is transcribed bicistronically together with *hpt* (Fig. 1A and Fig. 5A). The latter is essential and codes for the enzyme hypoxanthine phosphoribosyltransferase, which is part of the IMP biosynthesis pathway (via the salvage pathway of purine) (86). Operons with co-expressed genes often (but not always) contain functionally related proteins, which are also more likely to interact with each other (87–91). Thus, we speculate that *oloz0137* also has some function in the above pathway. However, only targeted experiments will reveal its function.

*oloz4542* appears to form a polycistronic unit with its downstream genes *ftsJ* and *hflB* (Fig. 1B and Fig. 5B). *ftsJ* codes for a 23S ribosomal methyltransferase (34) involved in cell division. *hflB* encodes a protease that carries out the proteolysis of various substrates (92, 93). The *hflb*/*ftsJ* operon was found to be regulated by a heat shock promoter in E. coli K-12 (94). Our data suggest that a transcript comprising all 3 genes is controlled by a promoter upstream of *oloz4542* under non-stress conditions in EHEC EDL933. It is conceivable that if required the 2 annotated genes are targeted for increased expression using the heat shock promoter. Interestingly, the protein product of *oloz4542* was predicted to be secreted and extracellular heat shock proteins are known to play an important role in the cellular stress response (95).

Finally, for *oloz5029*, we only have very limited information that allows for deducing a putative function. We do know, however, that the mother gene (*ehaG*) is a cell-surface virulence factor of many Gram-negative bacterial pathogens. EhaG is responsible for adherence, cell aggregation, and biofilm formation (35). Whether *oloz5029* may also have some function in virulence must remain open.

An interesting finding, however, was the emergence of shorter proteins that appeared alongside the full-length proteins in the Western blots for each OLO protein tested (Fig. 6). While a common interpretation would be the detection of degradation products (96), however, these shorter peptides could also represent protein isoforms (97, 98) expressed from alternative internal start codons, as recently shown for various bacterial proteins (20). Indeed, suitable alternative start codons are present at corresponding genetic positions (Fig. S1) supporting this hypothesis.

### Overprinting could create novel overlapping orphans.

BLAST analysis (Fig. 8) revealed that full-length OLO-homologs only occur in Escherichia/Shigella, indicating orphan genes. An apparent scenario would be an emergence through overprinting (63) after the split of the Escherichia/Shigella clade. Interestingly, many of recently characterized overlapping genes are taxonomically restricted and were suggested being orphans (21, 22, 24, 25). Orphans presumably code for non-essential, specialized functions that benefit the particular organism. In comparison to evolutionary older genes, orphans tend to be shorter, more disordered, and weakly expressed (28, 99). All these properties apply to the newly discovered genes, providing further evidence that they may indeed be evolutionary young.

### Conclusion.

In the past, bacteriological research was rarely focused on the identification of long overlapping genes. Neglecting the existence of such genes *a priori* prevented their discovery at a larger scale and indeed only very few such genes have been reported for bacteria. Most of these are even only serendipitous by-catch of other studies (11–14). Our findings highlight the fact that overlapping genes should not be dismissed easily. Assumedly, several more such fascinating genes are present in bacterial genomes than known to date (10).

## MATERIALS AND METHODS

### List of bacteria and incubation conditions.

Oligonucleotides are listed in Table S1 and bacterial strains and plasmids are listed in Table S2.

**(i) Creation of translationally arrested mutants.** EHEC genomic DNA, up- and downstream of the targeted mutation site, was amplified with primers overlapping at the position to be mutated (*oloz0137*: primers 1 + 4 and 2 + 3, *oloz4542*: primers 5 + 8 and 6 + 7, *oloz5029*: primers 9 + 12 and 10 + 11). A subsequent PCR incorporating the outer, non-overlapping primers (*oloz0137*: primers 1 + 2, *oloz4542*: primers 5 + 6, *oloz5029*: primers 9 + 10) was performed on the resulting amplicons to generate a complete gene fragment. The final fragment was then cloned into the plasmid pMRS101 (100), the high-copy *ori* was removed by NotI restriction digestion and modified plasmids were transferred to E. coli CC118λpir. Plasmids for which the correct sequence was confirmed by Sanger sequencing (Eurofins) were transferred to E. coli SM10λpir. For mating, a mix of EHEC EDL933 Nal^R^ (spontaneous Nal^R^ mutant) and E. coli SM10λpir containing the modified pMRS101 was plated on LB-agar and incubated for 24 h at 30°C. Cells were resuspended and EDL933 carrying the integrated plasmid were selected on LB-agar with 30 μg/mL streptomycin and 20 μg/mL nalidixic acid. Integration of the plasmid at the desired locus was confirmed by PCR. Cells were grown in LB to OD_600_ = 0.8 and plated on modified LB-agar; sodium chloride had been omitted but 10% sucrose was added in order to obtain selection plates for the double cross-over mutants (i.e., lacking *sacB*). Positive clones for translationally arrested mutants were identified by PCR using primers 13 + 14 (*oloz0137*), 15 + 16 (*oloz4542*) and 17 + 18 (*oloz5029*) and sequenced accordingly.

**(ii) RNA sequencing and ribosome profiling.** RNA-Seq and RP data of E. coli O157:H7 EDL933 (standard conditions, i.e., LB, 37°C, aerobic) were taken from Landstorfer, Simon (36). Reads were visualized as sum signal of 2 biological replicates in Artemis 17.0.1 (101) (Wellcome Sanger Institute).

**(iii) Individual growth curves.** For single strain growth, the translationally arrested mutants and EHEC wild type were grown separately in LB at 37°C. Overnight cultures of mutants and wild type were diluted to OD_600_ = 0.0003 with LB supplemented with 30 μg/mL nalidixic acid.

Next, 200 μL of the diluted cell culture were filled into the flat-bottom wells of a 96-well microtiter plate (Greiner Bio One). The plate was incubated at 37°C and 500 rpm for 9.5 h. OD_600_ was measured immediately (t_0_) and every 30 min during incubation (Victor^3^, PerkinElmer). OD_600_ values of each mutant and of the wild type were measured in biological triplicates. For each biological replicate, the mean for four samples was calculated after subtracting the zero control.

**(iv) Competitive growth assays.** To perform competitive growth assays, overnight cultures of wild type EHEC (not genetically modified) and each a translationally arrested mutant (both Nal^R^) were diluted to OD_600_ = 1 and mixed equally. Cells were pelleted by centrifugation (9000 × *g*, 3 min) from 500 μL of the mixture. The supernatant was discarded and the pellet was stored at −20°C (t_0_ control sample). A second aliquot of the mixture was diluted 1:300 and used to inoculate 10 mL LB supplemented with 30 μg/mL nalidixic acid down to OD_600_ = 0.0003. After bacteria were grown for 18 h at 37°C and shaking at 150 rpm, OD_600_ was measured and 500 μL of the cell culture was pelleted by centrifugation as before. Both pellets (t_0_, t_18 h_) were resuspended in sterile water, using 500 μL per 3 units of OD_600_ measured. A PCR was conducted using 1 μL of the aforementioned suspension as template using primers 19 + 20 (*oloz0137*), 21 + 22 (*oloz4542*), and 23 + 24 (*oloz5029*), respectively. PCR products were purified (GenElute PCR clean up kit, Sigma-Aldrich) and sequenced by Sanger sequencing (Eurofins) with the corresponding sequencing primer (*oloz0137*: 19, *oloz4542*: 21, and *oloz5029*: 23). The peak heights of the fluorescence signal in the sequencing chromatogram at the mutated gene locus were used to determine the competitive index CI (3). The CI was defined as the mutant-to-wild type ratio of the 18 h sample divided by the respective ratio of the t_0_ control sample: $\mathrm{CI}=\;(mt_{18\;h}/wt_{18\;h})/(mt_{t0}/wt_{t0})$. Each competitive growth experiment was conducted in triplicates.

**(v) Competitive growth with plasmid complementation.** For plasmid complementation, either the OLO wild type or the OLO mutant sequence was cloned into EcoRI and NcoI sites of pBAD/myc-HisC. Primers 66 + 67 were used for *oloz0137*, 68 + 69 for *oloz4542*, primers 70 + 71 for *oloz5029* and primers 72 + 73 for the *oloz5029* inverted sequence control. Plasmids were transformed into the respective EHEC EDL933 translationally arrested mutant and sequenced (Eurofins) with primer 31. Translationally arrested mutants carrying pBAD/myc-HisC (with either the wild type or mutant sequence of the respective arrested gene) were grown competitively against each other as described in the previous section. Protein expression was induced with 1.2% L-arabinose. Plasmids of the t_0_ and t_18 h_ sample were isolated and used as template in a Q5-PCR with primers 31 + 32. The resulting PCR products were sequenced with primer 74 and mutant CI was determined as described above.

**(vi) Overexpression and Western blots.** The plasmid pBAD-SPA (3, 102) was modified for protein expression as follows. The OLO-coding sequence (without its stop codon) was cloned into the vector in frame with the C-terminal SPA-tag using the restriction enzymes HindIII and NcoI in case of *oloz0137* and *oloz4542*, using primer pairs 25 + 26 and 27 + 28, respectively, or EcoRI and NcoI in case of *oloz5029*, using primers 29 + 30. The sequence of each construct was verified by Sanger sequencing (Eurofins) with primer 31. Modified plasmids (pBAD-OLO-SPA; i.e., carrying the SPA-tagged OLO frame in question) were transferred into E. coli Top10. For protein overexpression, 15 mL LB with 120 μg/mL ampicillin were inoculated 1:100 using an overnight culture of E. coli Top10+pBAD-OLO-SPA. Protein expression was induced at an OD_600_ = 0.3 by adding 0.002% arabinose. Samples of 1 mL were taken at time points 0, 1, 2, 3, 4 h, and after overnight incubation (about 16 h). All samples were measured for OD_600_ and diluted to OD_600_ = 0.3. Next, samples were centrifuged (16,000 × *g*, for 2 min at RT) and each pellet was boiled in 50 μL 3× sodium dodecyl sulfate (SDS) buffer (0.2 g SDS, 0.004 g Brilliant blue G, 4 g glycerol, 2 mL 1 M Tris pH 6.8) for 10 min at 95°C. A Tris-Tricine-SDS-PAGE according to Schägger (103) was used to separate proteins in the prepared samples with the following changes: 4% stacking gel, 0.6 mL acrylamide 40% bisacrylamide, 1.5 mL 3× gel buffer (3 M Tris, 1 M hydrochloric acid, 0.3% SDS), 3.9 mL water, 9 μL TEMED, 90 μL 10% ammonium persulfate (APS; Carl Roth); 16% running gel: 4.8 mL acrylamide:bis-acrylamide 40% 29:1 (Carl Roth), 4.05 mL 3× gel buffer, 1.2 mL 85% glycerol (Merck), 1.95 mL water, 8 μL TEMED (Carl Roth), 80 μL 10% APS. Next, 8 μL of each sample lysate was run on the gel for 2.5 h at 35 mA per gel in cathode buffer (1 M Tris, 1 M Tricine, 1% SDS, at pH 8.25) and anode buffer (1 M Tris, 0.225 M HCl, at pH 8.8). As positive control, 1 μL of SPA-tagged E. coli gluthatione S-transferase was used and prepared in 3× SDS buffer as described above. The PageRuler Prestained Protein Ladder (Thermo Fisher Scientific) was used for protein size determination. Proteins were blotted on a PVDF membrane (PSQ membrane, 0.2 μm, Merck Millipore) at 12 V for 20 min in a semi-dry blotter. Subsequently, the membrane was washed in 3% trichloroacetic acid (TCA, Carl Roth) and in water, each for 5 min, followed by overnight incubation in 25 mL 1×TBS-T (10 mM Tris pH 8; 0.15 M sodium chloride; 0.5% Tween20, Sigma-Aldrich) with 1.25 g of nonfat milk powder at 4°C. After blocking, the membrane was washed thrice for 10 min with TBS-T. Subsequently, 10 μL of AntiFLAG M2-AP antibody in 10 mL TBS-T were applied to the membrane and incubated for 1 h while shaking. Superfluous antibodies were removed by washing the membrane 6-times for 5 min with TBS-T. After a 1-min equilibration with the reaction buffer (0.1 M Tris, 4 mM MgCl_2_, at pH 9.5), SPA-tagged proteins were visualized by incubation in 10 mL reaction buffer supplemented with 100 μL NBT (50 mg nitro-blue tetrazolium [Applichem] in 1 mL 70% dimethyl formamide [Sigma-Aldrich]) and 125 μL BCIP (20 mg 5-bromo-4-chloro-3-indolyl phosphate, Carl Roth). As soon as protein band were clearly visible while gentle shaking, the reaction was stopped with 3% TCA (about 10 to 60 s after incubation).

**(vii) Test for promoter activity using pProbe-NT.** The promoterless plasmid pProbe-NT (104) was used to test for the promoter activity of about 300-nt long fragments upstream of each proposed OLO start codon. The promoter fragment was cloned into the vector using the primer pairs 33 + 34 (*oloz0137*), 35 + 36 (*oloz4542*), and 37 + 38 (*oloz5029*), respectively. The resulting plasmid was transformed into E. coli DH5α. The correct sequence was verified by Sanger sequencing using primer 32. For promoter activity testing, overnight cultures of E. coli DH5α+pProbe-NT-PromOLO were used to inoculate 25 mL LB supplemented with 30 μg/mL kanamycin at a ratio of 1:100. In parallel, E. coli DH5α and E. coli DH5α+Probe-NT were cultivated as reference and negative control, respectively. When reaching OD_600_ = 0.6, 1 mL bacterial culture was pelleted by centrifugation (12,000 × *g*, 2 min). The pellet was resuspended and washed once with 1 mL PBS. After washing, 200 μL of the suspension were aliquoted four times in a black 96-well microtiter plate. Fluorescence was measured for each of the 4 technical replicates using a Wallac Victor^3^ (PerkinElmer Life Science) at an excitation of 485 nm, and emission 535 nm, using a measuring time of 1 s. The background fluorescence of the reference sample was subtracted and the mean and standard deviation of 3 biological replicates was calculated.

### Quantification of OLO mRNA by qPCR.

Translation of the OLOs was analyzed in early and late exponential, as well as in stationary phase. Therefore, 100 mL LB + 30 μg/mL nalidixic acid were inoculated with 1 mL overnight culture of EHEC EDL933 Nal^R^ and incubated at 37°C. Aliquots of the cultures were taken at OD_600_ = 0.3 (25 mL), 1.5 (5 mL) and 3.0 (3 mL), respectively. Each aliquot was centrifuged (9,000 × *g* for 5 min at 4°C). The supernatant was discarded, and the remaining pellet was frozen in liquid nitrogen and stored at −80°C. RNA was extracted from the frozen samples with TRIzol (Thermo Fisher Scientific). Cell pellets were resuspended in TRIzol (2.4 mL for the sample of OD_600_ of 0.3 and 4 mL for samples of OD_600_ of 1.5 and 3.0, respectively). About 300 μL 0.1-mm Zirconia beads (Carl Roth) were added. Bead beating with a FastPrep (MP Biomedicals) disrupted the cells (three times, 6.5 m/s for 45 s, and cooled on ice for 5 min between each run). For each 1 mL of TRIzol, 200 μL chloroform was added; the sample was vortexed and incubated for 5 min. Centrifugation (12,000 × *g*, 4°C, 15 min) separated the organic from the aqueous phase. RNA in the aqueous phase was precipitated with 500 μL isopropanol and 1 μL glycogen (Thermo Fisher Scientific) for 30 min and subsequently pelleted via centrifugation (12,000 × *g*, 4°C, 10 min). The pellet was washed twice with ice-cold 70% EtOH, dried at RT and resuspended in 100 μL molecular-microbiology water. Remaining DNA was digested with TURBO DNase (Thermo Fisher Scientific) according to the manufacturer’s instructions. Absence of DNA was verified by PCR using *Taq*-polymerase (NEB) and 16S rRNA primers 39 + 40. RNA integrity was verified on a 1.5% agarose gel. RNA transcripts of the OLOs were analyzed by qPCR. EHEC EDL 933 RNA was reverse transcribed with Superscript III reverse transcriptase (RT, Thermo Fisher Scientific) according to manufacturer’s instructions using gene specific primers 41, 42, 43, and 44 for *oloz0137*, *oloz4542*, *oloz5029*, and *cysG*, respectively. As a negative control (no-RT), samples without reverse transcriptase were used. For RT-PCR, 1 μL of the cDNA-preparation was used as template in a *Taq*-PCR with 30 cycles for *oloz0137* and *oloz4542*, and 35 cycles for *oloz5029*, respectively. Primers have been used as indicated in Fig. 8. PCRs with the no-RT controls confirmed primer specificity in each case, and PCRs with genomic EHEC DNA as template-validated primer binding (data not shown). The qPCR mix contained 10 μL SsoAdvanced Universal SYBR green Fast Mix (BioRad), 1 μL of each forward or reverse primer, 5 μL cDNA (from above) and 3 μL water. The two-step amplification reaction was conducted with 98°C initial denaturation for 30 s, 40 cycles of denaturation at 98°C for 15 s and annealing/elongation at 56.9°C for 30 s. A melting curve analysis was conducted for the final products (60°C to 95°C in steps of 0.5°C, each for 5 s). Primer efficiency was tested at 56.9°C, using 100 ng, 10 ng, 1 ng, 0.1 ng, and 0.01 ng genomic DNA of EHEC EDL933 as template. The following efficiencies were determined: 95% for *oloz0137* (primers 45 + 46), 93% for *oloz4542* (primers 47 + 48), 102% for *oloz5029* (primers 49 + 50), and 94% for *cysG* (primers 51 + 52), respectively. Samples taken at the respective OD_600_ (i.e., 0.3, 1.5, and 3.0) were measured in biological and technical triplicates. Amplification specificity was verified by using a no-RT control. The quantity of each OLO mRNA relative to the reference gene *cysG* was determined using the ΔΔCq-method according to Pfaffl (105): Primer efficiency E was determined and ΔCq-values were calculated by subtracting the Cq values of the tested genes from the Cq value of *cysG*. The expression ratio was calculated as E^-ΔCp^.

### Bioinformatic analysis.

**(i) Promotor determination.** Sequences upstream of the start codons were screened with BPROM (41) to identify σ^70^ promoters with a minimum LDF score threshold of 0.2.

**(ii) Identification of Shine-Dalgarno sequences.** The region 30 nt upstream of the start codon was analyzed for a Shine-Dalgarno sequence motif according to Ma, Campbell (106).

**(iii) BLAST analysis.** Homologous proteins were detected with the Basic Local Alignment Search Tool (BLAST); specifically, tblastn was used here (55). The amino-acid sequence of the OLOs was used to query the NCBI nt/nr database and results were filtered to match records with at least 70% percent identity, minimum 90% query coverage and an e-value ≤ 0.001.

**(iv) Phylogenetic tree construction**. Multiple sequence alignments (MSA) of OLO-sequences were constructed using MUSCLE implemented in MEGA11 (107). A neighbor-joining phylogenetic tree was created for representative species based on the alignment and bootstrapped 100 times.

**(v) Protein prediction.** Hypothetical OLO protein structure and function were predicted using PredictProtein (44) implementing the programs RePROF (108), TMSEG (109), Meta-Disorder (110), ROFbval (111), ProNA (112), DISULFIND (113), and Conseq (85) in one pipeline. AlphaFold predictions were created with AlphaFold (45) via ColabFold (114).

**(vi) Stem loop prediction.** RNA secondary structure was predicted using the RNAfold web server (115).
