# Supplementary material for: Three Novel Antisense Overlapping Genes in E. coli O157:H7 EDL933
Source: Microbiol Spectr. 2022 Dec 19;11(1):e02351-22. doi: 10.1128/spectrum.02351-22 (PMC9927249; doi:10.1128/spectrum.02351-22)
Supplement: Supplemental file 4 — Supplemental material. Download spectrum.02351-22-s0004.pdf, PDF file, 2.2 MB [file spectrum.02351-22-s0004.pdf]

Supplementary files

Experimental Evidence for Three Novel Antisense Overlapping Genes in *E. coli* O157:H7 EDL933

Franziska Graf[1], Barbara Zehentner[2], Lea Fellner, Siegfried Scherer[1,2] and Klaus Neuhaus[1,2*]

[1] Core Facility Microbiome, ZIEL – Institute for Food & Health, Technische Universität München Weihenstephaner Berg 3, 85354 Freising, Germany.

[2] Chair for Microbial Ecology, TUM School of Life Sciences, Technische Universität München Weihenstephaner Berg 3, 85354 Freising, Germany.

*email for correspondence: neuhaus@tum.de

Files included here

Table S1: List of oligonucleotides

Table S2: List of bacterial strains and plasmids

Figure S1: Genomic context of oloz0137, oloz4542 and oloz5029

Figure S2: Predicted protein structure of oloz0137, oloz4542 and oloz5029

Figure S3. AlphaFold predicted protein structure

Figure S4: Growth curves for EHEC wild type and each translationally arrested mutant

Figure S5. Phylogenetic tree and sequence alignment of the OLOs.

Figure S6. Transcription and translation of the putative overlapping gene ORFs conserved in EHEC Sakai

Files not included here

Data S1: Fasta of all *oloz0137* homologs found (provided as csv).

Data S2: Fasta of all *oloz4542* homologs found (provided as csv).

Data S3: Fasta of all *oloz5029* homologs found (provided as csv).

Supplementary Table S1. List of oligonucleotides used in this study.

primer no.	name	sequence (5' -> 3')
1	Z0137+403F-SpeI	ATACTAGTGACGAAATGCCGCAAGAGCG
2	Z0137+860R-ApaI	GCGGGCCCAGTCGTTGGCGATTTGTACG
3	Z0137+630RmutS	TGGCGTGTTTTAACTTGAGGTTG
4	Z0137+630FmutS	CAACCTCAAGTTAAAACACGCCA
5	Z4542+8F-SpeI	ATACTAGTGCAAAAAATACGATGAATCT
6	Z4542+445R-ApaI	GCGGGCCCGTGGAATACCTGTAAGAATT
7	Z4542+238Fmut	CCTGTAATGTTCAAGTCATCGGTA
8	Z4542+238Rmut	TACCGATGACTTGAACATTACAGG
9	Z5029+570F-XbaI	GATCTCTAGAGAGTATTGCTCTTGGCGACA
10	Z5029+1092R-BamHI	GATCGGATCCCATCGTAACTGTCAGACTTG
11	Z5029+825F-mutS	CGCCATAGGTCAAGGCAACAAAGC
12	Z5029+825R-mutS	GCTTTGTTGCCTTGACCTATGGCG
13	Z0137+159F	CGGTCTTGAGCCGGGCGAAC
14	Z0137+1120R	CTGGTGGGTCTGCTGCGTGG
15	Z4542+136F	TGGAGCTGGCAGAACGCTTCTT
16	Z4542+588R	CATCGTTGGCGATGACGAAGCT
17	Z5029+444F	CTCAATGGCCCTCGGCCGTT
18	Z5029+1250R	AAGTACAGCGAGCGCAGCCC
19	Z0137+692R	CTGGACGAGTAAGTGTGAAGTTGC
20	Z0137+568R	GATCTGGATGTTACTGCCACCAAC
21	Z4542+153F	CGCCACCGAAGATCGCGAAACTAA
22	Z4542+285R	ACTTTAGGATAATCTTAGCGTGGC
23	Z5029+736F	AGCATGGCAATGGGGTTCGGAAGC
24	Z5029+856R	CATTACCCAGAGCGATGGCATCCAC
25	OLOZ0137Western_F-NcoI	GATCCCATGGATGGCATTTTTATTTGTGGT
26	OLOZ0137Western_R-HindIII	ATCAAGCTTCGCGTGGAAACGCGGGCAGAA
27	OLOZ4542Western_R-HindIII	ATCAAGCTTCTTTGGTGATATATGGGAGAT
28	OLOZ4542Western_R-EcoRI	GAGCGAATTCTTTGGTGATATATGGGAGAT
29	OLOZ5029Western_F-NcoI	GATCCCATGGCTGTCAGACTTGATCGCGCC
30	OLOZ5029Western_R-EcoRI	GAGCGAATTCTTCAAAAGCATTGGGTAAAT
31	pBAD Primer F	CAGAAAAGTCCACATTGATT
32	pProbe-NT-R	CGTATGTTGCATCACCTTCA
33	OLOZ013-400F-SalI_PromF	GGCTGTCGACCAGGTATCTCATGAAGTCGA
34	OLOZ0137-19R-EcoRI_PromR	GTCGGAATTCGCAACTTCACACTTACTCGT
35	OLOZ4542-220F-SalI_PromF	GGCTGTCGACGTAAGAATTACCCAATACTC
36	OLOZ4542-19R-EcoRI_PromR	GTCGGAATTCGTGCTTTATCGCCCAACTAA
37	OLOZ5029-338F-SalI-PromF	GGCTGTCGACCTTCGACATCGTCATTGGTA
38	OLOZ5029-0R-EcoRI_Prom2R	GTCGGAATTCTGCGGATTTAGACAATACCG
39	rrsHF	AATGTTGGGTTAAGTCCCGC
40	rrsHR	GGAGGTGATCCAACCGCAGG
41	OLOZ0137_qPCR_RT_R	GAAACGCGGGCAGAAAGTTA
42	OLOZ4542_qPCR_RT_R	TATATGGGAGATGGCGGTAG
43	OLOZ5029_qPCR_RT_R	CATTGGGTAAATTGTCTATT
44	cysG+241R_RT	TGGTTAAGCGCGTCA
45	Z0137+568F	GATCTGGATGTTACTGCCACCAAC

46	OLOZ0137_RTPCR_F1	ATTTGTGGTTGGCGTGTTTC
47	OLOZ4542_qPCR_F	CATCCGCTCAAGCCAGTTGT
48	OLOZ4542_qPCR_R	TAGTTTCGCGATCTTCGGTG
49	OLOZ5029_qPCR_F	CATTTTCAGCGGCTTCGGTC
50	OLOZ5029_qPCR_R	AGTATTGCTCTTGGCGACAC
51	cysG Ec fw	TTGTCGGCGGTGGTGATGTC
52	cysG Ec rev	ATGCGGTGAACTGTGGAATAAACG
53	OLOZ0137_TermRTPCR_F	AGTAACATCCAGATCACGCA
54	OLOZ4542_RTPCR_F	AGTACTCAGATTCATCGTAT
55	OLOZ5029_RTPCR_F	AGCAATACTCATAATCTCAG
56	OLOZ0137_TermHPT_1F	CAAAGTGATTCTGCTGGACGAGTA
57	OLOZ0137_TermRTPCR_3R	TGGTTACAGGTCGTTAACCT
58	OLOZ0137_TermRTPCR_4R	CTTCGACCTGCAAATCTATT
59	OLOZ0137_TermRTPCR_5R	CAACTGACGTTTAGCGTTCA
60	OLOZ4542_TermRTPCR_5R	CTCATGTTTGGTGCCATATC
61	OLOZ4542_TermRTPCR_6R	CTGGAACACCTTCACTACAA
62	OLOZ4542_RTPCR_Term7	TCACTCAAGGGATTAACCTC
63	OLOZ5029_TermRTPCR_2R	TACTTTTATGAACACCAGTG
64	OLOZ5029_TermRTPCR_3R	CAGTAAGCTGTTAATTTCTG
65	OLOZ5029_TermRTPCR_4R	TTATAACAAGGAATAGCGAG
66	OLOZ0137-400F-NcoI	GATCCCATGGCAGGTATCTCATGAAGTCGA
67	OLOZ0137+517R-EcoRI	GAGCGAATTCTGGTTACAGGTCGTTAACCT
68	OLOZ4542-332F-NcoI	GATCCCATGGCTGTAAACTCACCTATTGCT
69	OLOZ4542+518R-EcoRI	GAGCGAATTCTCATTCCCGGTTTAAAGAGT
70	OLOZ5029-338F-NcoI	GATCCCATGGCTTCGACATCGTCATTGGTA
71	OLOZ5029+1001R-EcoRI	GAGCGAATTCCTGTTAATTTCTGCGCTGGT
72	OLOZ5029-338F-EcoRI	GAGCGAATTCCTTCGACATCGTCATTGGTA
73	OLOZ5029-338F-EcoRI	GATCCCATGGCTGTTAATTTCTGCGCTGGT
74	pBAD-C+193F	CGTCACACTTTGCTATGCCATAGC

Supplementary Table S2. List of strains and plasmids used in this study.

strain	characteristics	reference
Escherichia coli O157:H7 EDL 933	wildtype, outbreak strain, obtained from Collection de l'Institute Pasteur (CIP106327)	Perna et al., 2001
Escherichia coli O157:H7 EDL933 Nal^R	Spontaneous nalidixic acid resistant clone	Fellner et al., 2015
Escherichia coli O157:H7 EDL933 Nal^RΔOLOZ0137	mutant, translational arrest of OLOZ0137	this study
Escherichia coli O157:H7 EDL933 Nal^RΔOLOZ4542	mutant, translational arrest of OLOZ4542	this study
Escherichia coli O157:H7 EDL933 Nal^RΔOLOZ5029	mutant, translational arrest of OLOZ5029	this study
Escherichia coli Top10	Strain for general cloning	Invitrogen
Escherichia coli CC118	May carry suicide plasmids with pir-dependent ori	Manoil and Eckwith, 1985
Escherichia coli SM10λpir	May carry suicide plasmids with pir-dependent ori	Miller and Mekalanos, 1988
pBAD/mc-HisC	derivate of pBR322, *araBAD* promoter,<br>myc C-tag and his-tag fusion, Amp^R	Invitrogen
pBAD/SPA	derivate of pBR322, *araBAD* promoter,<br>C terminal SPA-tag fusion, Amp^R	Zehentner *et al.*, 2020
pMRS101	Suicide plasmid for gene knock-out or mutation knock-in	Sarker and Cornelis, 1997
pProbe-NT	Promoter probe plasmid	Miller *et al.*, 2000

Figure S1: Genomic context of *oloz0137*, *oloz4542* and *oloz5029*. Sequences of the overlapping open reading frames (OLOs), their mother genes and putative co-transcribed genes are given. Color code:

OLO start alternative start stop -10 box -35 box TSS SD sequence co-transcribed terminator
mother gene start stop

OLOZ0137

agctttattaacagattccgcgaatgaatcgttttgctggtatactgcgtgtcttgcgctttgttgcggtgccaaaacctgcccgtgcgaagtgattt
gtttttaaatcatatggttagagatatgaaacatactgtagaagtaatgatccccgaagcggagattaaagcgcgtatcgccgaactgggtcgtc
agattactgagcgttacaaagacagcggcagcgatatggtgctggtgggtctgctgcgtggctcatttatgtttatggcggacctgtgccgtgaag
ttcaggtatctcatgaagtcgactttatgaccgcctccagctacggtagcggcatgtccaccacccgtgatgtgaaaatcctcaaagatctggatg
aagatatccgtggcaaggatgtgctgattgttgaagatatcatcgactcggggaatacactgtcgaaagtgcgtgagatcttaagcctgcgcgaa
ccgaagtcgttggcgatttgtacgctgctggataaaccgtcccgtcgtgaagtgaacgtcccggtagaatttatcggtttctcgatcccggatgagt
ttgtggtgggttacggcattgattacgcacagcgttaccgtcatctgccgtatatcggcaaagtgattctgctggacgagtaagtgtgaagttgccg
gatgtgttgcatccggcatggcattttatttgtggttggcgtgtttcagcttgaggttggaaatcccgtgacggtaacgttgctcaagggtttcgcg
gttggtggcagtaacatccagatcacgcagcaagccgtcgtgaatgccataagcccagccgtgaatggtaactttctgcccgcgtttccacgctga
ttgcataatggtggagtggcccaggttatacacctgttccatgacgttcagttcacacaaggtatccagacggcgctcttgcggcatttcgtcgagc
aatgagctatgtttgaaccagatatcgcggatatgcagcagccagttgttgataagccccagttccgggttttcaactgcggcttgtacgccgccg
caaccgtagtggccacagataataatgtgttcaacttcgagtacatccactgcatactgaaccacggaaaggcagttcaggtcggtgtgaatgac
caggttagcaacattacggtgaacaaagagttcgcccggctcaagaccggttaaacgttctgcaggaacgcgactgtcggaacatccaatccat
agaaagcgcggtttttgcgcttgtgccagtttctcaaaaaacccgggatcctcttccaccagcatttttgaccatagtgcattgttgctgatgagtgt
atctatgtctttcatggaggttaacgacctgtaaccaaataattacgtttggctaatatagggcaactccgggacgatttaaaccacagataaagt
gtaagaacgtaaggtaagtaaaaatttatgaccattgcactggaacttcaacagcttaaaaaaaactatccaggcggcgttcaggcgcttcgtgg
aatagatttgcaggtcgaagc

OLOZ4542

gtcagtagactacgcccttgaatgaacagcggattgacgagactgtaccacaatgggcccaagccgcatgcgccaagtagttggcctgctcctcg
gaaacctgatggtaacagcggtcgtaacaatcttcgcggaccaagaagggtcgggcgaccgcctgcatgtagcgctgcacggtagtccagtagt
tagacgccacgcagctatcagagtataaagtactctcgtagaaagtagcgtacgtcttataggtataacagccagtcttcgactgcgcgtgctaat
atcaatgcgagttagtcacgaaagtgaaggaccagctactatgcacgtcaaagtagcctgtatacaaagcgaaagcgatgcgatgccggctcct
tgtgaagtggaagaagaaggcgcatgtcgtcacctgggttaaagagtctcttcggggtgactcagtggtacaatgcacggtccaaccagcgttga
aattatagcaacctgcgtggccatctttgtacaaggccgggcatctactagagaagacggtctgctggtggcatgtcgcatctttagaccgaaagg
tcaaagactgccgaccgcgactaacgcagcgggagtccgttcttctttcagtgtgggtcgcgtgctggcgcaccctattagcagtgaaacacgtgg
cctagcacgaggccgtggtccgctggctattagcgtactgggcgaagcaccattcggcagctaagaaagacgcgaaggcagtagtggtacctcg
ctgcaaggcgtgggtagtactaaaacagaaagcgaaagagcttgagttggtagctgtgttgcgcaaacaacggtgctcgtcgcttgtcacggcg
aagcaagtggtccaagcggtccagtcgtggcctctttggtcctcatggtgcccgttactaacggcgcagctatagcccacggttaccatgcgctgc
gtacacttggaagtcctagacgagtgctggcgcttgtagaccgttcggttggtgttggactgccagctttgccggtcctgcgtcgtcgcgacccagc
tcttgcaggcctgccaatcagcgccgctattgctactatggaagcaatggaagcttcggtaggtagagttggtcgtagaccaagtctcagacaagt
gcaagtagcactggtggcgggtctggacgcggtgcgaccgccggatgccgcagctaaagtagctatttctactacgtgccacggcgaaagaagc
ggacaagcttgtacagtgcttgtgccctacgtgggtgtggctgcttgtaaagatgcttcagtcttggcctctatcatttcttgccttgaaagcgaagc
ggacgttagcgaaagcggtcgtcgcaaaatggccatgggcctcctggctggtagtcctgcggaaagccctagaatggcggctcaaagaccttcg
ccgagccgagcgcgtctatgagttggtcaagtcgttgaagaagaaaacgaagcagcgtcgggcgttgcagtcgtttccagcaaaactagactag
aaggcagtcgtacgcgcgaaatgagaatggtttgctgtaccgtggaaacggtggcggcgggacgtaaactgcgtacttcttctaggtctgtggtt

agtcgttgtcgtagcccttggtcctctacttctatcttcggtcgtccgacccaagaagtccgccaagtggctgttggaactgcaagaaccagttgtcc
aataggtcattaaagcctaggacttggccttacattcaccatattgccaatgatagaaagaaccattgcaactaaagtgcaggcaactatgcgcg
aagtgcttggaccagcaataactggagaacatccttccatctcattaggtggaatgccggtaatctgagcgacccgggtttcgagaccttatgact
gtagtcgtgttgccgttactggtcggtctcataatccaaaaagcggtacagtgagttccctaattggagaataatgttgacacaatttttgtcgcag
tcctatgagatataggtcgtagaaagtttgaaagcagactttagagggccaatcccaaatgcgggccagcgatgttatatgtgaagggcgcttgc
acgtgctcttctcaggccgaatgcttgaaactggaagcatttgtccctcgcttaaagggaatctatgagtagctttggaagcgggaccttgtggaa
gtgatgttttgacggtggaccgcgattatgtagtgctgtgtaaagatcgcggtcaaggtggtctatgtaccgtgcccccctataggtggcggccaca
aggcgagtacaaaccacggtatagcctgtactgttggacctgaaacgacagcggttgcgcgaggtcgtcacgaaagtagtggtcaagtagtgct
tttagcgggacttctttcaggtgtggttgctatcctaggtatccattttctagcgttcgctactacgccggaaacggcggttaaacccactggtgtat
aacactggttggtgggcctcgtcgtggttccagctgttggcagtaagggccaaatttctcaaacagtgaaacgacataaagtagttcaaatttggt
ccgtgcccttgcattggggaaaaagacacggacaacttgtataaatagcgatttcacaaggacttcggtcgccgacctcgaccgtcttgcgaaga
atggacagtaaattgaaagggtacccctattgagtagcattggttaacgcatttaaaaatgcgcggataaaccactatataccctctaccgccatc
ttactgggcaaaagttagggttgcattcgtttttatgctacttagactcatgatttgtttttgtcgtggactttccagaccgtgtaggcgagttcggtc
aacaagacgaaccgtcattaccaaactggcttccccacgaccggctctaacttgttcgcaatctcgtggtacttgagtagttccacttttagcggtg
gcttctagcgctttgattttggaactagcaccttcgatagcacgcgctttggccgcggacattacatgtccagtagccattttgcgaccacgaaata
gcgggttgatttcttgcattttagagcgacggtgcgattctaataggatttcaatgtgtgtaaacgacacattttgctccccaaaaggcg

OLOZ5029

tacttgttttataaatttcaatagaccttgggacgctgtcccttaatatgacaatggtcgctttgccgtttttcggcaccgttctttagacccgcgtcat
tcgacaattaaagacgcgaccaacgcccaccttacaacagcagcaaaccccgtaaccgcttacggcccttactgttgccagtcccacaactaatg
ccatcacctagtcgaccgctgccgacccaacgatatccgtttccccgctttcgtttatgaaaatacttgtggtcaccaaggtcatgacgacacccaa
tactgcgatatcgacttccggttatatcgagacggtagcccagtttttgggtacgctaaccaccacgtagttaccggaaaccccaatcacgttatag
tcttccgctatcttcatatcgtgacccacggagaagcataagtaacccggttatgagttaccgggagccggcaataagttttcgtaacccatttaac
agataacgatacccccctgagaaggtttcgccttcctcgtttgcggtaacgggaccctttacggtgatttcgatgactctaatactcataacgagaac
cgctgtggcggttacgcagttttcgcataagttaccgcgaccctcgttcatcgcagcgtagacttcttttgcgataacgctatccacgactctggctt
cggcgacttttacgttgacgataaccgttattacgcttccgttttccctgattatcgtaccgttaccccaagccttcggaacggctatttcagttatga
tagcgtaatcctttaccgtcggtccgagaccgtctattacgttagcggtatccggtcccgttgtttcgactaccgcacctacggtagcgagacccatt
accatcggtcagctctccgaatttgtggtaacggaatccgtgtcggtcattacgttgaccactattctcagaacgcgaaccatcattatcgtcacgg
ttgccataattgagacagcgcgacccgcgtctaaggtaacgcctaaatctgttatggcagagacagccgttatcaagtaattttgcgttctagcaa
ttacactttttaccgcgctagttcagactgtcaatgctacggtaattaccaagtgtcgaaatacggtaatcgctgagccatcgtttttccgaacctcc
tccccgacgtcatctacaactactgccatgacaatgtcgtggttggatgttaaatttttttaccatcgtttttattgcatccccgacgcgagcgacatg
aactacttttgtgggacgttaccctggtttggtttccgtttatgtcgcgacgagtaccatgatcatcgggttgacggtcgcattagtggctacaacgc
ctaccgtgctaaagtcggaggtcattcctacgccaattgccaagggttgactttcgatggttactgctacagcttcggttgtggcggttatagcgat
gattatggtcgttgtaacggtgcttatgccgtttataacggtggttatggtggttatagtggttggactgcctaaggcaaccactggaagtccgact
acgggacgagaccttgctttgatttttccgtaagtcacgtcgagtgccggtcctatggtggtcgttttagtggttgcaatttctacggctggactgcc
gactgtcgtgactacgacaattgccgagagtcgacttttggtggttgctacgacaccgctgcttatggtggttatagcggttattgtgaaggttata
acggtgattgtggtggttgtagagattggactgactctgccactgattagaaccactcctacgcgactttaccctattcctgttaccacataagtgcc
gtcgagtaccgtggctctggtggtcgttttagtggttgcaatttctaccgctggactgctgaccgtcgtggctacggcaattgccgagagtcgacttt
tggtggttgctacggcaccgctgcttatggtggttatagcggtgattgtggtggttgtagagattagactgactctgccactgattagaaccactcct
acgcgactttaccctattcctgttaccacagaagtgacgtcgagtaccgttgttatggcggtcgttttagtggttataggacctgccgtgtcagtgac
gttggtcaaggctacggtaattgccatcggtcgaaatactgaattcgtcgttatagcggtggatgaagccgccgttacgaagacacttatgactgc
cacacaaatggccaggttggatgttttagccactttgtttaataatattgcagccgctacgtgaccgacgctaattgaggagtaaatcgtgcagag
agccgctacgagacgaaaccctacggtggcgtccatttaagtcacggtttgtgccatgattaccactgcgttcgcactagtgactacagcgtctac
cactttaaagtctgaggtcaagactgcgtcatttgccgagtgttgaggtgccgcactcgtcaatacaacaactacgcgacccccccaccacggcttc
agttacgtctgccgtggtagtgacgcggctgcatgtggtaacgattacgactaatgctattacagccactacgggacttacgatagctatggtgag
aactgctgcgagacgagaccctacgcctgcggccacttttaccacgtaaatcgcggcgagtgccttttctattttgacggtcacattagtgattgca

gcgattgccacgttagagacgacggtcgtcgctgcgctaattgccgagtgttgagatatggtggttattcatgtagcgactacgcgacccaccact
gcgtcttcagttgcgactgccgtggtagtggcgtggctgaatgtggtaacgcttgcggctcatgttgttgcagccactgcgggacctacgcgaact
actattgcgggacgacaccctactctgacggttaccgccacgacctcggatgttacggtcggtactgccatttcggtcgtagtagtgattacagcg
attaccgtcataatcactcctgtcatggctacgtcacttgccaagagtcaacttacgctgcttatactactaactcgtcttgtgggtttaatagttagt
cgagcgaccattgtggctgcgttggatataggttcttttgccacgcccataattgatacacgcatgattgctgctgccgaatcgcaagttgctgcgg
tcgcgtgtcccacaaccgcgatgtcgatatccaatattgagacagcggtttccgctatcgtcgcatcgataaccagtcccgtcgatgtcgctgcaac
tatgcccatagcgggacccatcgtcgagacaaaggtcggctcactaacggtttccgagggcactgtggtcgtattgccttttaccgcaacaataac
caatgctgtggtgcctaccgcttgacgagccacgtaacagatagccactactgccatttatagcagtttagtagttgcatcggctaccaaggcttcg
ggtactgcggcaatgccaagcagttaacgtcttacgctaaccacgccagcgttggtgcggctgatttatgaaggtgcgattaagttgccttcttcta
agtgaccgtcacccttgactgagcgaccgttacccacgcttttggtagcacttaccgctatttccataaccatagccaataccacggatgcacctgc
gcttacgtgaattgccgtaacggtaaccatcgttacgcgttcagtaagtacagttgtcataacgctatccattaccaagatgctggtgagcaccgc
gagtttggttaatatggcggatgttgtacctgcgtggcgtcttgagacagccacttaagagtcagccatcacgcctaccagttgcagtctagtgatt
gcagcgtcgtccaagccgactatggctacgccagttgcacccagtcaactttcattgcctacgcgtccaaagggtcttatgggtctcgtaatgattg
gacctattagcccattgcttagaactaagtgcgcagtggttatagcttttgccataaccgctatagcagtggtggccatcgtggttcatgaagttctg
gttatggctaccacatctacggtcgcgcgtcccatttctatcgcagcgctaaccaaggccgaggtaacgacggcgactgttgtcgcagcgagacc
catgtcccagacaccgttggcttcttttatgctagagacatccaaggagatgattggttgcagcatagtggttgcatcgacgtccatttttacgatgg
ctacgacaattgcaccgtgtcaacttcagaaggcttcgaccgccacatgcaatgctgtggtttcgactaccaagatagctgatatcgttatagtgg
gagccaccgccgttgccgccatgctgagcatagtcgttgcagaggcgaccgcagttgttgttgctgcaccacttaatgcgcgtcaacttcgtttcgc
acgtcctttgcttcgttatgtggctagtcgcttaccaactctacctattgtttgacagattttgactttcgttcaactcgccaccatagcgaagacgtt
accgttactggccagacggcgtccgaatgtgaggtccacggtcgtaccggagataaccaccgccatgaatgttgccacttagccgtcaacgaaat
ccacatagctaccactcgcggttaccagcaacccagatgtttaatgttccatcatggttatcggtcccacttatgaggcggcgtgagccacggcca
taagtcaccatt

Figure S2. PredictProtein predicted protein structure. Indicated features of the overlapping open reading frames *oloz0137* (**A**), *oloz4542* (**B**) and *oloz5029* (**C**) were predicted with PredictProtein (Bernhofer et al., 2021).

A

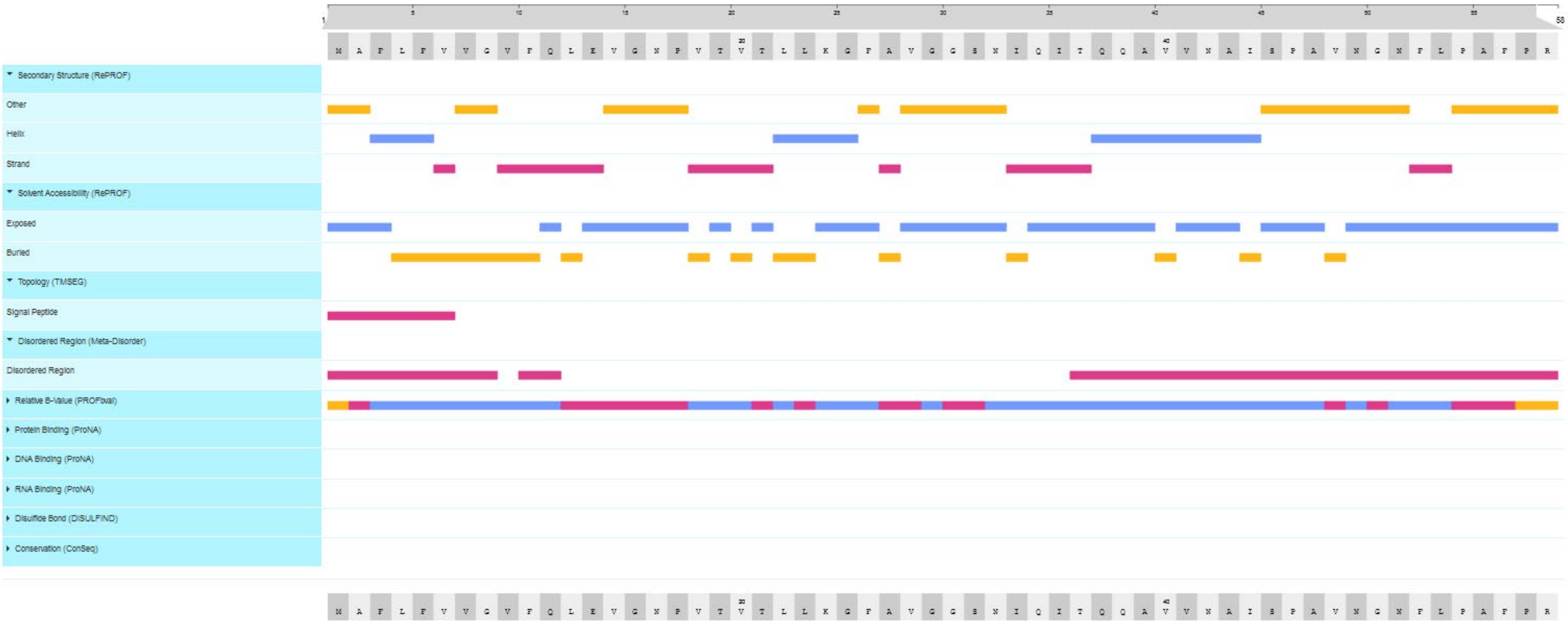

B

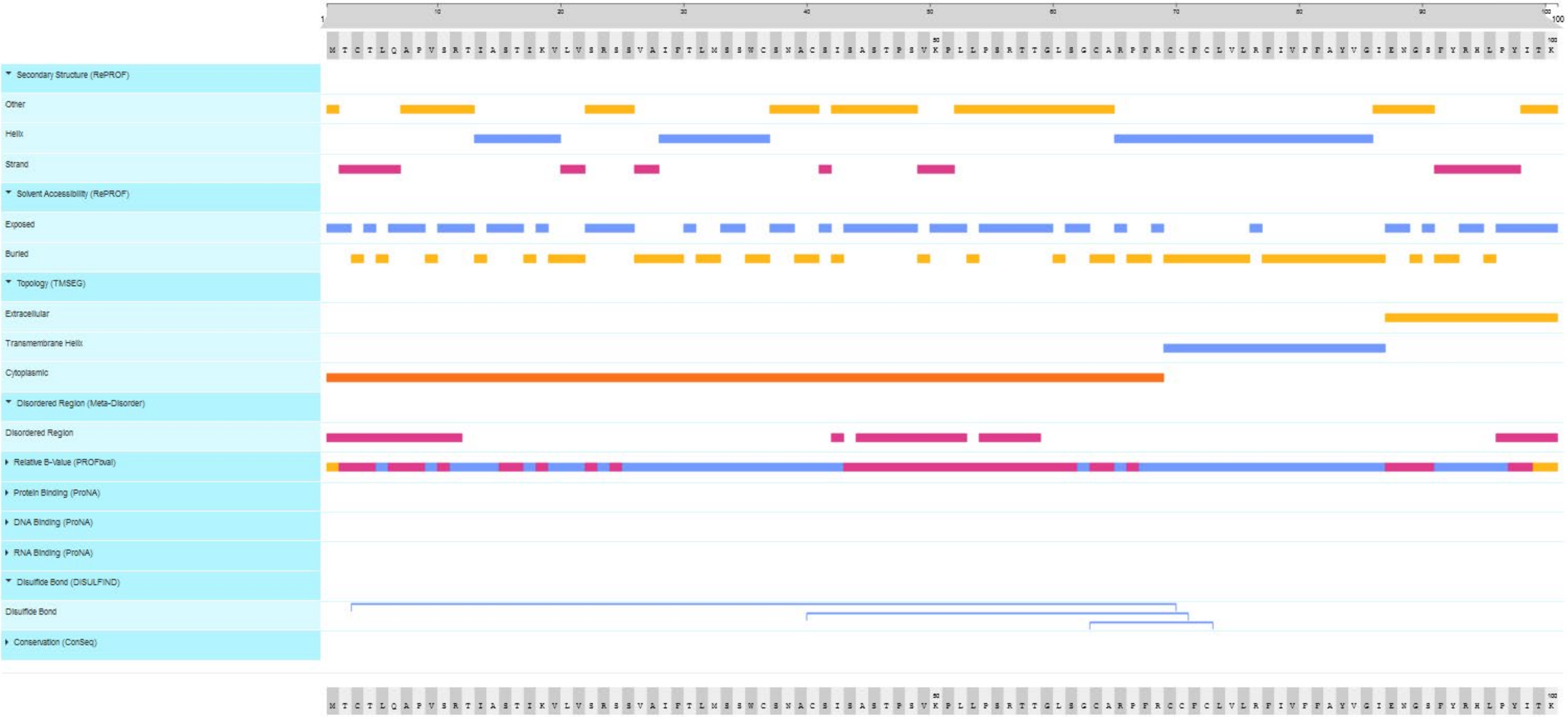
Secondary Structure (RePROF)
Other
Helix
Strand
Solvent Accessibility (RePROF)
Exposed
Buried
Topology (TMSEG)
Extracellular
Transmembrane Helix
Cytoplasmic
Disordered Region (Meta-Disorder)
Disordered Region
Relative B-Value (PROFbval)
Protein Binding (ProNA)
DNA Binding (ProNA)
RNA Binding (ProNA)
Disulfide Bond (DISULFIND)
Disulfide Bond
Conservation (ConSeq)
M T C T L Q A P V S R T I A S T I K V L V S R S S V A I F T L M S S W C S N A C S I S A S T P S V K P L L P S R T T G L S G C A R P F R C C F C L V L R F I V F F A Y V G I E N G S F Y R H L P Y I T K

C

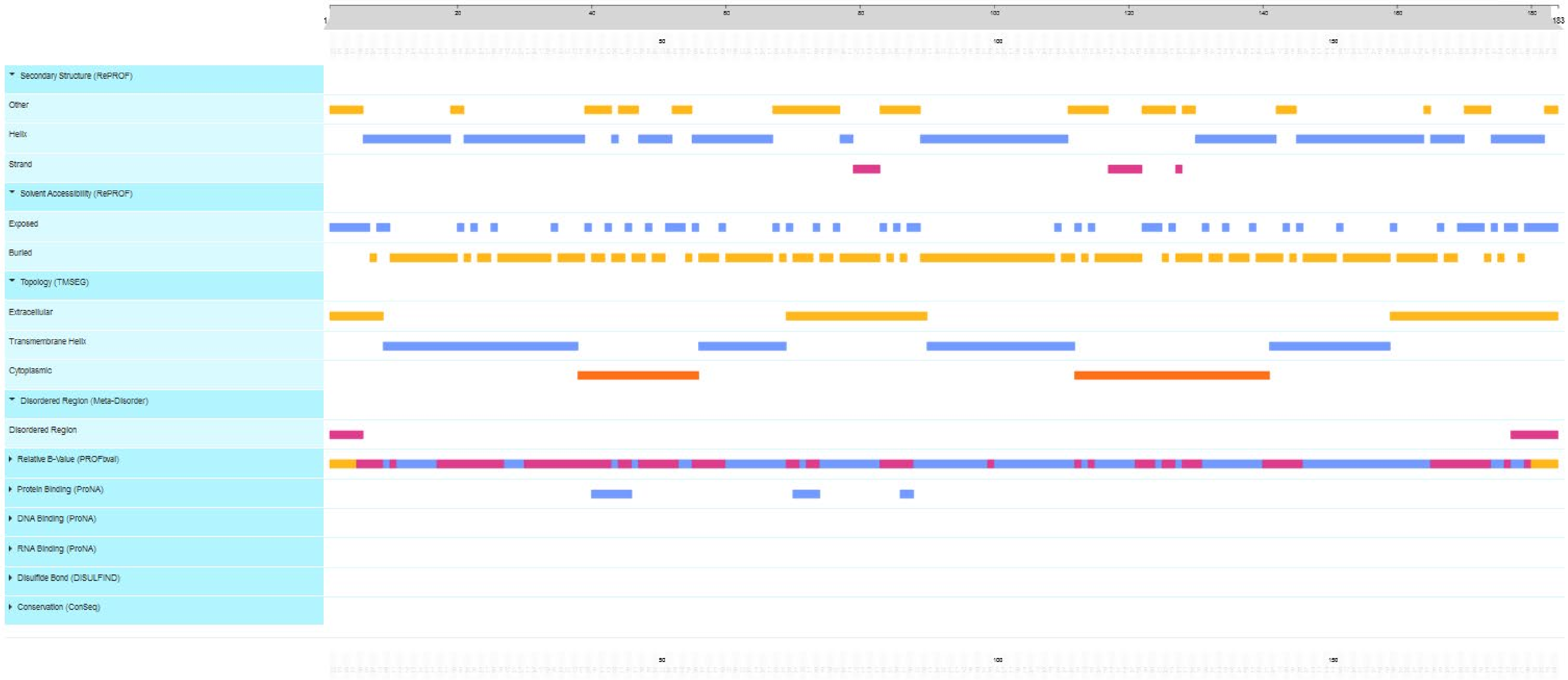
1
20
40
60
80
100
120
140
160
180
183
50
100
150
Secondary Structure (RePROF)
Other
Helix
Strand
Solvent Accessibility (RePROF)
Exposed
Buried
Topology (TMSEG)
Extracellular
Transmembrane Helix
Cytoplasmic
Disordered Region (Meta-Disorder)
Disordered Region
Relative B-Value (PROFbval)
Protein Binding (ProNA)
DNA Binding (ProNA)
RNA Binding (ProNA)
Disulfide Bond (DISULFIND)
Conservation (ConSeq)

Figure S3. Alphafold predicted protein structure. Indicated features of the overlapping open reading frames *oloz0137* (**A**), *oloz4542* (**B**) and *oloz5029* (**C**) were predicted with AlphaFold (Jumper et al., 2021). lDDT: local Distance Difference Test.

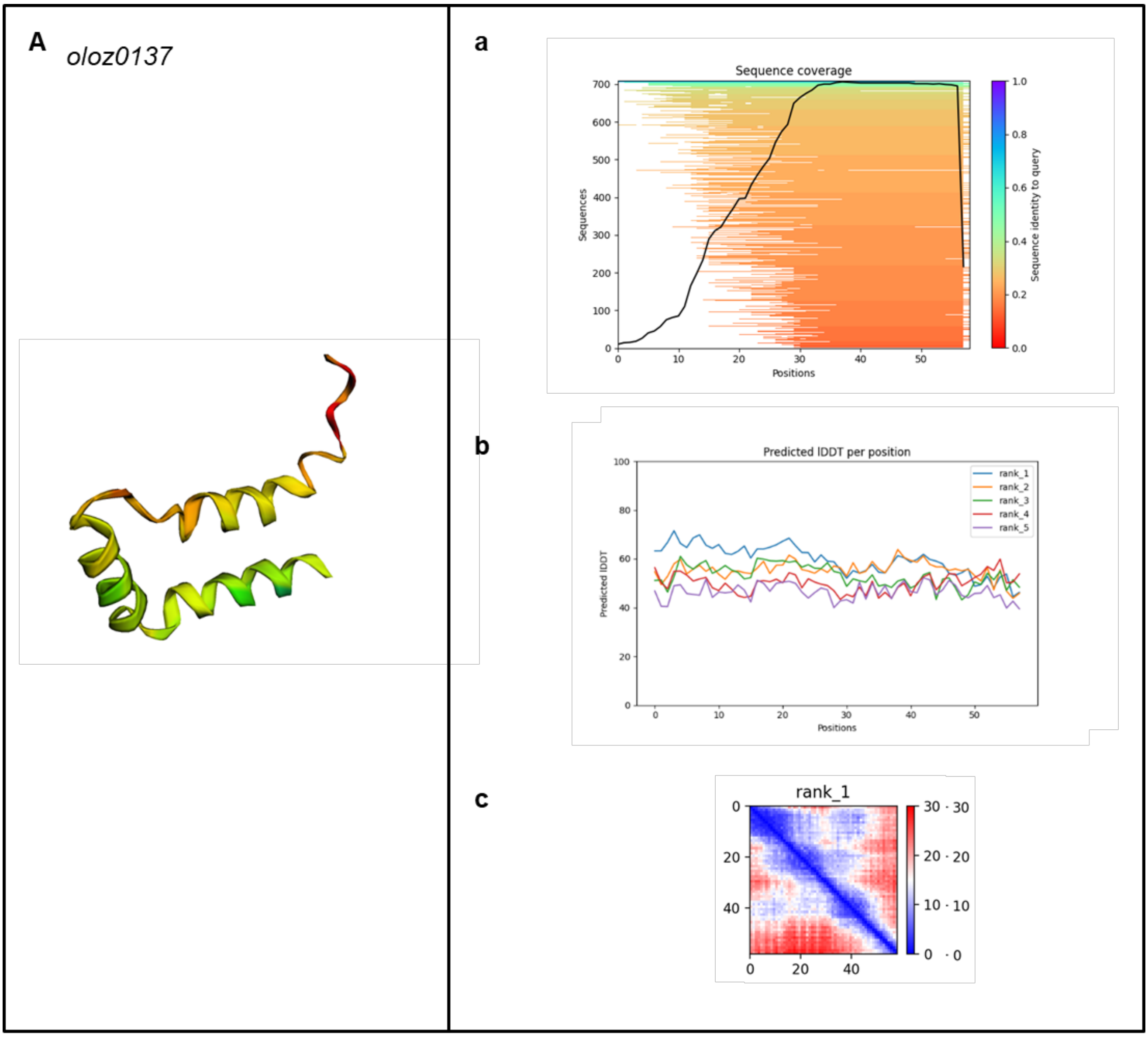

B *oloz4542*

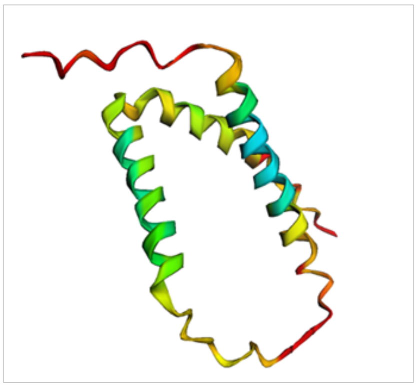

a

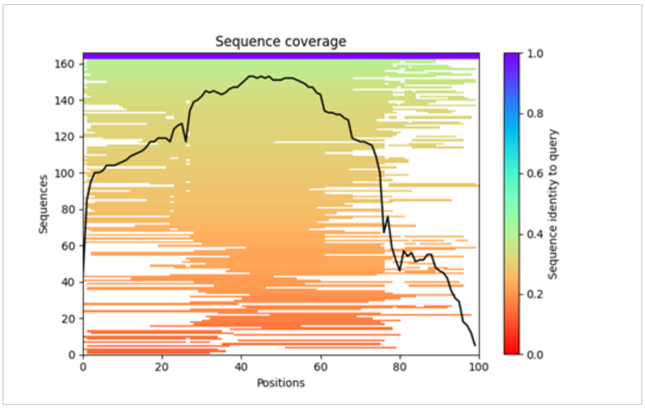

b

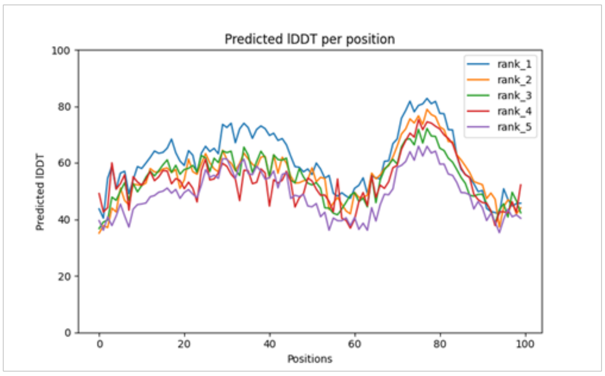

c

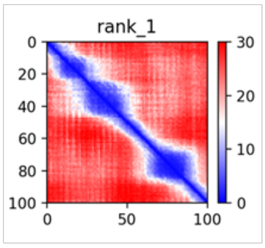

c *oloz5029*

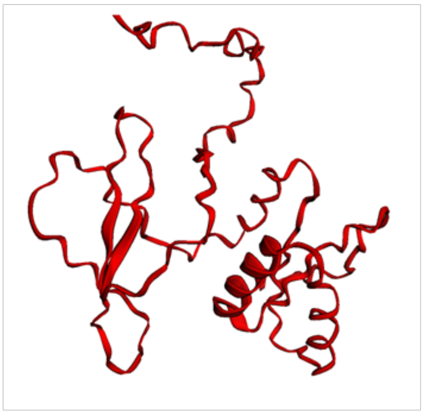

a

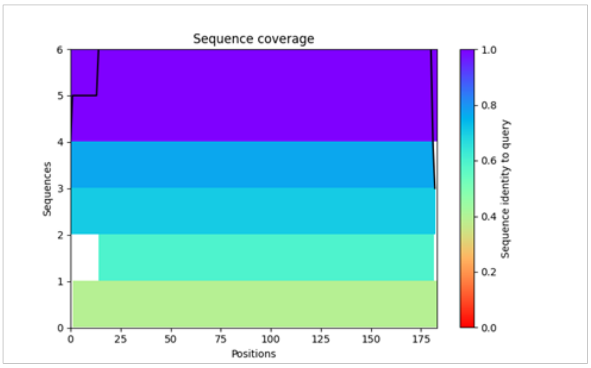

b

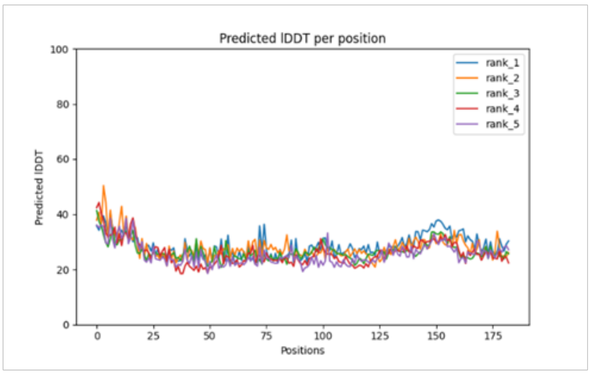

c

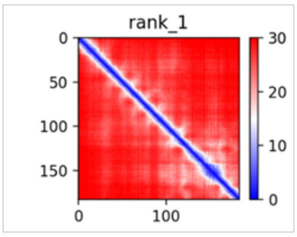

Figure S4. Growth curves for EHEC wild type (wt) and each translationally arrested mutant (Δ*oloz0137*, Δ*oloz4542*, and Δ*oloz5029*). Each strain was grown separately in a microtiter plate under standard conditions (LB, 37 °C, shaking at 150 rpm). Mean OD_{600} values and standard deviations of three biological replicates are shown.

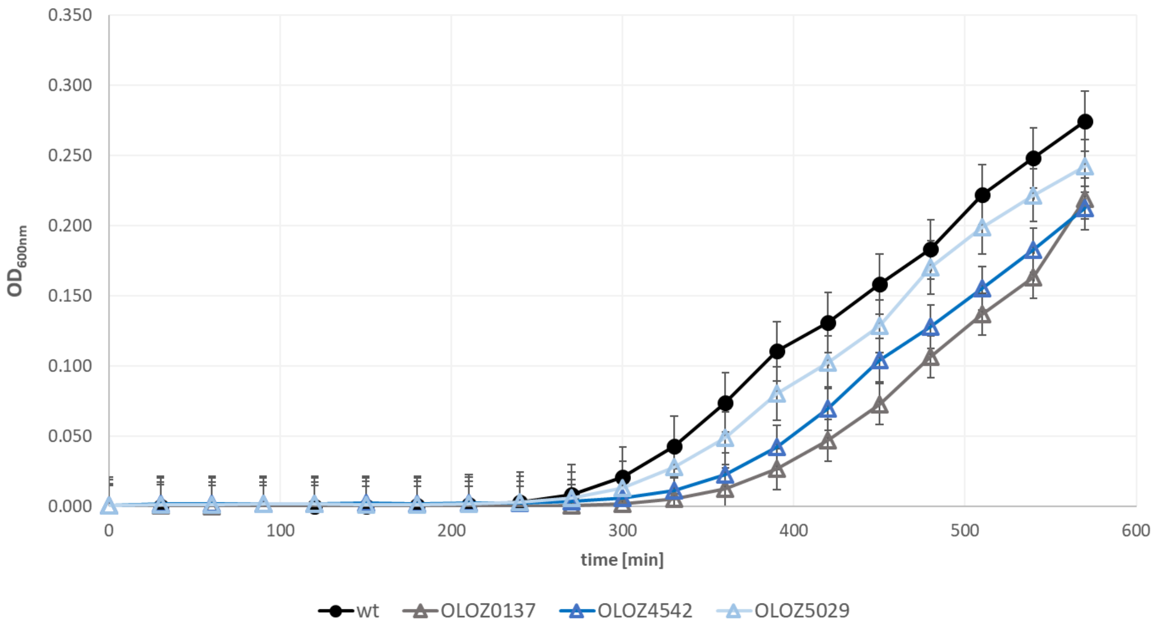

Figure S5. Phylogenetic tree and sequence alignment of the OLOs. In MEGA11 (Tamura et al., 2021), a multiple sequence alignment was conducted and a phylogenetic Neighbor-joining tree was constructed for representative species. **(A)** *oloz0137*, **(B)** *oloz4542*, and **(C)** *oloz5029*.

A

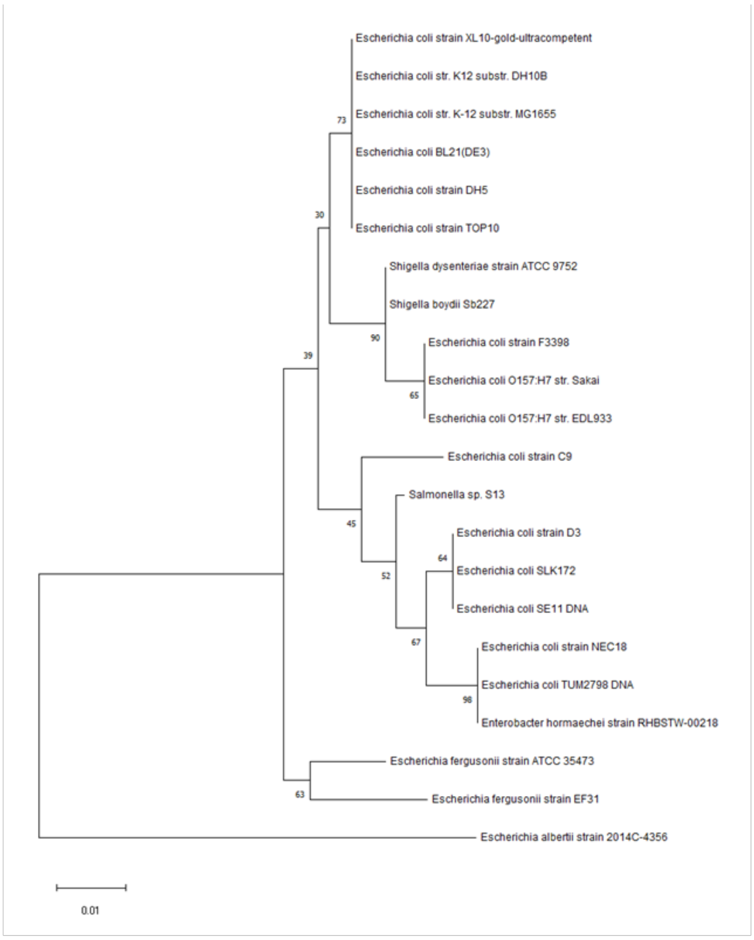

1. Escherichia coli strain F3398	MAFLFVVGVFQLEVGNPVTVTLLKGFAVGGSNIQITQQAVVNAISPAVNGNFLPAFPR*
2. Escherichia coli O157:H7 str. Sakai	MAFLFVVGVFQLEVGNPVTVTLLKGFAVGGSNIQITQQAVVNAISPAVNGNFLPAFPR*
3. Escherichia coli O157:H7 str. EDL933	MAFLFVVGVFQLEVGNPVTVTLLKGFAVGGSNIQITQQAVVNAISPAVNGNFLPAFPR*
4. Shigella dysenteriae strain ATCC 9752	MAFLFVVGVFQLEVGNPVTVTLLKGFAVGGSNIQITQQAVVNAIGPAVNGNFLPAFPR*
5. Shigella boydii Sb227	MAFLFVVGVFQLEVGNPVTVTLLKGFAVGGSNIQITQQAVVNAIGPAVNGNFLPAFPR*
6. Escherichia coli strain DH5	MAFLFVVGVFQLEVGNPVTVTLLKGFAVGGGNIQITQQAVVNAVGPAVNGNFLPAFPR*
7. Escherichia coli strain TOP10	MAFLFVVGVFQLEVGNPVTVTLLKGFAVGGGNIQITQQAVVNAVGPAVNGNFLPAFPR*
8. Escherichia coli BL21(DE3)	MAFLFVVGVFQLEVGNPVTVTLLKGFAVGGGNIQITQQAVVNAVGPAVNGNFLPAFPR*
9. Escherichia coli str. K-12 substr. MG1655	MAFLFVVGVFQLEVGNPVTVTLLKGFAVGGGNIQITQQAVVNAVGPAVNGNFLPAFPR*
10. Escherichia coli strain XL10-gold-ultracompetent	MAFLFVVGVFQLEVGNPVTVTLLKGFAVGGGNIQITQQAVVNAVGPAVNGNFLPAFPR*
11. Escherichia coli str. K12 substr. DH10B	MAFLFVVGVFQLEVGNPVTVTLLKGFAVGGGNIQITQQAVVNAVGPAVNGNFLPAFPR*
12. Salmonella sp. S13	MAFLFVVGVFQLEVGNPVTVTLLKGFAVGRCDIQITQQAVVNAVGPAVNGNFLPAFPR*
13. Escherichia coli strain C9	MAFLFVVGVFQLEVGNPVTVTLLKGFAIGRCNIQITQQAVVNAVGPAVNGDFLPAFPR*
14. Escherichia fergusonii strain ATCC 35473	MAFLFVVGVFQLEVGNPVTVTLFKGFAVGGGNIQITQQTVVNAVGPAVNGDFLPAFPR*
15. Escherichia coli SE11 DNA	MAFLFVVGVFQLEVGNPVTVTLLKGFAVSRCDIQITQQAVVNAIGPAVNGNFLPAFPR*
16. Escherichia coli strain D3	MAFLFVVGVFQLEVGNPVTVTLLKGFAVSRCDIQITQQAVVNAIGPAVNGNFLPAFPR*
17. Escherichia coli SLK172	MAFLFVVGVFQLEVGNPVTVTLLKGFAVSRCDIQITQQAVVNAIGPAVNGNFLPAFPR*
18. Escherichia fergusonii strain EF31	MVFLFVVGVFQLEIGNPVTVTLFKGFAVGGGNIQITQQTVVNAVGPAVNGNFLPAFPR*
19. Escherichia coli strain NEC18	MAFLFVVGVFQLEVGDPVTVTLLKGFAVSRCDIQITQQAVVNAVGPAVNGNFLPAFPR*
20. Escherichia coli TUM2798 DNA	MAFLFVVGVFQLEVGDPVTVTLLKGFAVSRCDIQITQQAVVNAVGPAVNGNFLPAFPR*
21. Enterobacter hormaechei strain RHBSTW-00218	MAFLFVVGVFQLEVGDPVTVTLLKGFAVSRCDIQITQQAVVNAVGPAVNGNFLPAFPR*
22. Escherichia albertii strain 2014C-4356	MANLFVIDVLQLEVGNPVAVTLLQGFAVSGSDIQVTQ*TIVNAVGPAMNGHFLPALP

B

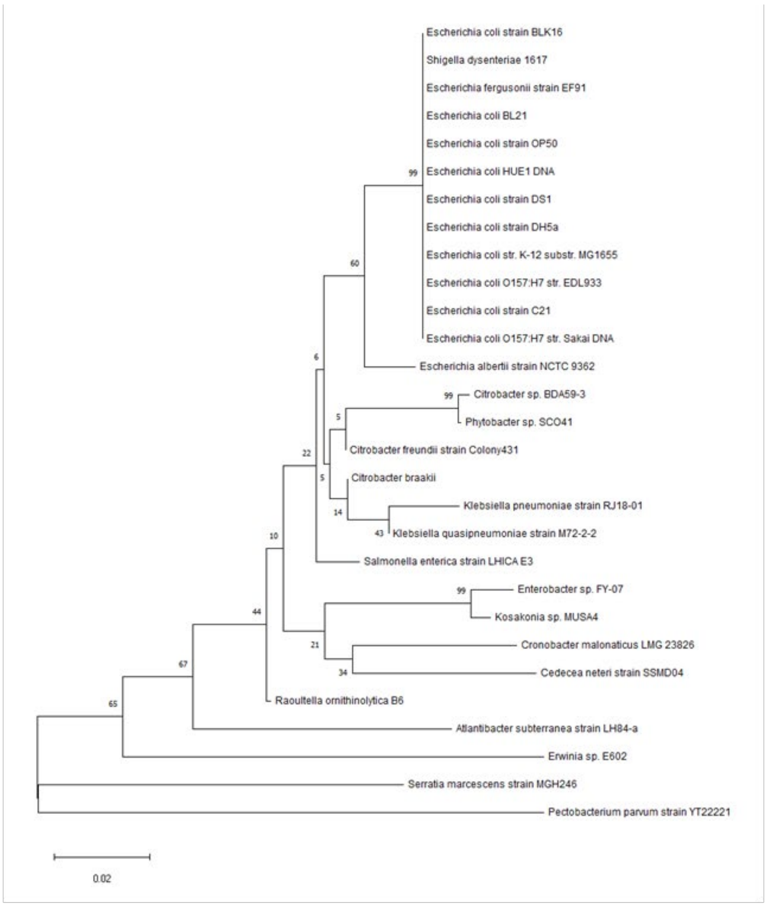
Escherichia coli strain BLK16
Shigella dysenteriae 1617
Escherichia fergusonii strain EF91
Escherichia coli BL21
Escherichia coli strain OP50
Escherichia coli HUE1 DNA
Escherichia coli strain DS1
Escherichia coli strain DH5a
Escherichia coli str. K-12 substr. MG1655
Escherichia coli O157:H7 str. EDL933
Escherichia coli strain C21
Escherichia coli O157:H7 str. Sakai DNA
Escherichia albertii strain NCTC 9362
Citrobacter sp. BDA59-3
Phytobacter sp. SCO41
Citrobacter freundii strain Colony431
Citrobacter braakii
Klebsiella pneumoniae strain RJ18-01
Klebsiella quasipneumoniae strain M72-2-2
Salmonella enterica strain LHICA E3
Enterobacter sp. FY-07
Kosakonia sp. MUSA4
Cronobacter malonaticus LMG 23826
Cedecea neteri strain SSMD04
Raoultella ornithinolytica B6
Atlantibacter subterranea strain LH84-a
Erwinia sp. E602
Serratia marcescens strain MGH246
Pectobacterium parvum strain YT22221
99
60
6
99
5
22
5
14
43
10
99
44
21
34
67
65
0.02

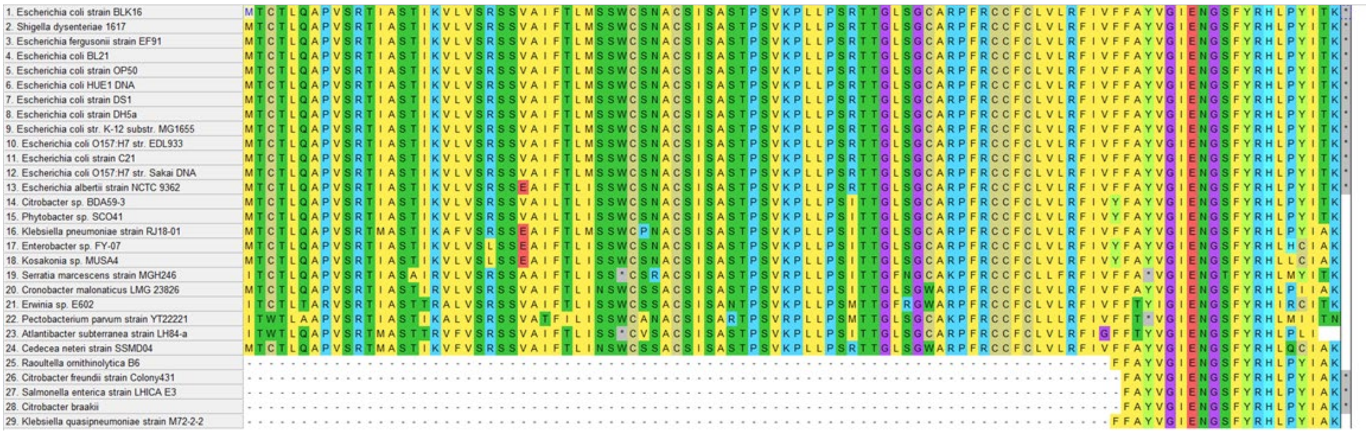
1. Escherichia coli strain BLK16
2. Shigella dysenteriae 1617
3. Escherichia fergusonii strain EF91
4. Escherichia coli BL21
5. Escherichia coli strain OP50
6. Escherichia coli HUE1 DNA
7. Escherichia coli strain DS1
8. Escherichia coli strain DH5a
9. Escherichia coli str. K-12 substr. MG1655
10. Escherichia coli O157:H7 str. EDL933
11. Escherichia coli strain C21
12. Escherichia coli O157:H7 str. Sakai DNA
13. Escherichia albertii strain NCTC 9362
14. Citrobacter sp. BDA59-3
15. Phytobacter sp. SCO41
16. Klebsiella pneumoniae strain RJ18-01
17. Enterobacter sp. FY-07
18. Kosakonia sp. MUSA4
19. Serratia marcescens strain MGH246
20. Cronobacter malonaticus LMG 23826
21. Erwinia sp. E602
22. Pectobacterium parvum strain YT22221
23. Atlantibacter subterranea strain LH84-a
24. Cedecea neteri strain SSMD04
25. Raoultella ornithinolytica B6
26. Citrobacter freundii strain Colony431
27. Salmonella enterica strain LHICA E3
28. Citrobacter braakii
29. Klebsiella quasipneumoniae strain M72-2-2

C

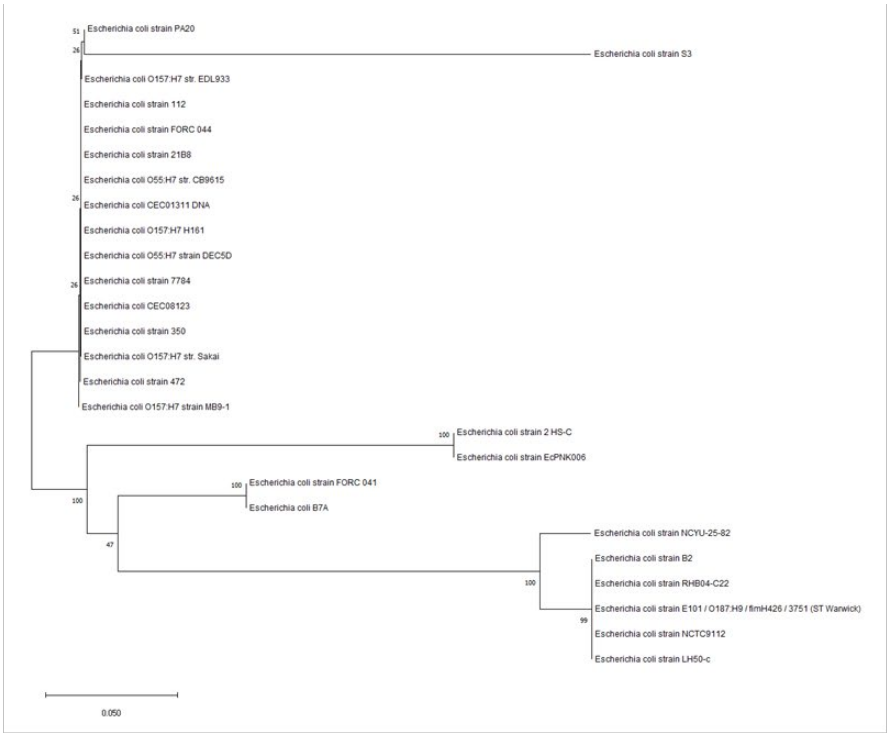
51
26
Escherichia coli strain PA20
Escherichia coli strain S3
Escherichia coli O157:H7 str. EDL933
Escherichia coli strain 112
Escherichia coli strain FORC 044
Escherichia coli strain 21B8
Escherichia coli O55:H7 str. CB9615
26
Escherichia coli CEC01311 DNA
Escherichia coli O157:H7 H161
Escherichia coli O55:H7 strain DEC5D
26
Escherichia coli strain 7784
Escherichia coli CEC08123
Escherichia coli strain 350
Escherichia coli O157:H7 str. Sakai
Escherichia coli strain 472
Escherichia coli O157:H7 strain MB9-1
100
Escherichia coli strain 2 HS-C
Escherichia coli strain EcPNK006
100
Escherichia coli strain FORC 041
Escherichia coli B7A
100
47
Escherichia coli strain NCYU-25-82
Escherichia coli strain B2
Escherichia coli strain RHB04-C22
100
Escherichia coli strain E101 / O187:H9 / fimH426 / 3751 (ST Warwick)
99
Escherichia coli strain NCTC9112
Escherichia coli strain LH50-c
0.050

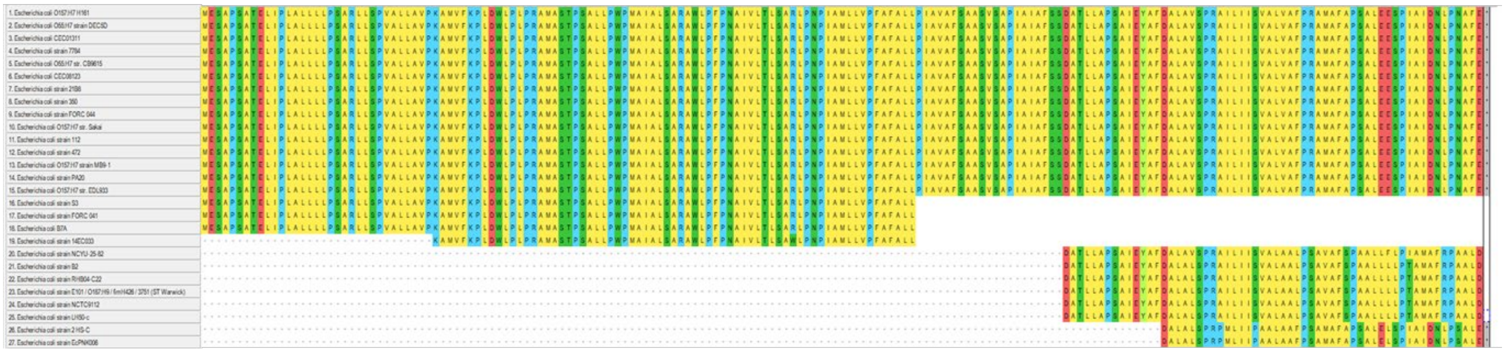

Figure S6. Transcription and translation of the putative overlapping gene ORFs conserved in EHEC Sakai (Hücker et al., 2017). Ribosome profiling and RNA sequencing data were obtained from Hücker *et al.* (2017). Top panels, transcription (RNA sequencing); bottom panels, translation (ribosome profiling). *E. coli* O157:H7 str. EDL933 was cultivated under standard conditions (LB, 37 °C) and harvested at the beginning of the early stationary phase. The sum signal of two biological replicates are visualized using Artemis 17.0.1 (Carver et al., 2012). **(A)** *oloz0137*, **(B)** *oloz4542*, and **(C)** *oloz5029*.

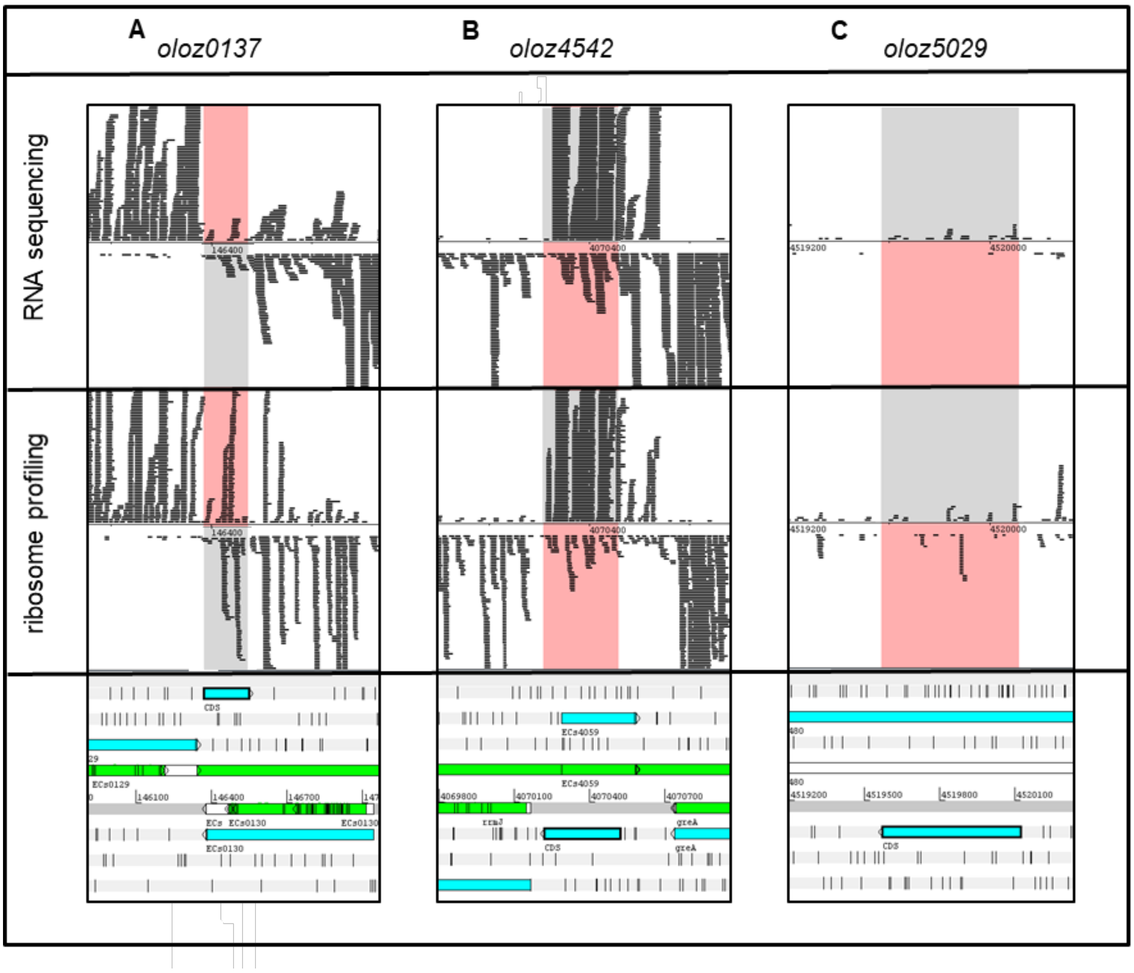

References

Bernhofer, M., Dallago, C., Karl, T., Satagopam, V., Heinzinger, M., Littmann, M., Olenyi, T., Qiu, J., Schütze, K., Yachdav, G., et al. (2021). PredictProtein – Predicting Protein Structure and Function for 29 Years. Cold Spring Harbor Laboratory.

Carver, T., Harris, S.R., Berriman, M., Parkhill, J., and McQuillan, J.A. (2012). Artemis: an integrated platform for visualization and analysis of high-throughput sequence-based experimental data. Bioinformatics *28*, 464-469. 10.1093/bioinformatics/btr703.

Hücker, S.M., Ardern, Z., Goldberg, T., Schafferhans, A., Bernhofer, M., Vestergaard, G., Nelson, C.W., Schloter, M., Rost, B., Scherer, S., and Neuhaus, K. (2017). Discovery of numerous novel small genes in the intergenic regions of the Escherichia coli O157:H7 Sakai genome. PLOS ONE *12*, e0184119. 10.1371/journal.pone.0184119.

Jumper, J., Evans, R., Pritzel, A., Green, T., Figurnov, M., Ronneberger, O., Tunyasuvunakool, K., Bates, R., Žídek, A., Potapenko, A., et al. (2021). Highly accurate protein structure prediction with AlphaFold. Nature *596*, 583-589. 10.1038/s41586-021-03819-2.

Tamura, K., Stecher, G., and Kumar, S. (2021). MEGA11: Molecular Evolutionary Genetics Analysis Version 11. Mol Biol Evol *38*, 3022-3027. 10.1093/molbev/msab120.
